# One-dimensional photonic crystal enhancing spin-to-orbital angular momentum conversion for single-particle tracking

**DOI:** 10.1038/s41377-024-01623-x

**Published:** 2024-09-26

**Authors:** Mingchuan Huang, Qiankun Chen, Yang Liu, Chi Zhang, Rongjin Zhang, Junhua Yuan, Douguo Zhang

**Affiliations:** 1 Advanced Laser Technology Laboratory of Anhui Province, Department of Optics and Optical Engineering, University of Science and Technology of China, Hefei, Anhui China; 2 Department of Physics, University of Science and Technology of China, Hefei, Anhui China; 3 Hefei National Research Center for Physical Sciences at the Microscale, University of Science and Technology of China, Hefei, Anhui China; 4 Hefei National Laboratory, University of Science and Technology of China, Hefei, China

**Keywords:** Interference microscopy, Nanophotonics and plasmonics

## Abstract

Single-particle tracking (SPT) is an immensely valuable technique for studying a variety of processes in the life sciences and physics. It can help researchers better understand the positions, paths, and interactions of single objects in systems that are highly dynamic or require imaging over an extended time. Here, we propose an all-dielectric one-dimensional photonic crystal (1D PC) that enhances spin-to-orbital angular momentum conversion for three-dimensional (3D) SPTs. This well-designed 1D PC can work as a substrate for optical microscopy. We introduce this effect into the interferometric scattering (iSCAT) technique, resulting in a double-helix point spread function (DH-PSF). DH-PSF provides more uniform Fisher information for 3D position estimation than the PSFs of conventional microscopy, such as encoding the axial position of a single particle in the angular orientation of DH-PSF lobes, thus providing a means for 3D SPT. This approach can address the challenge of iSCAT in 3D SPT because DH-PSF iSCAT will not experience multiple contrast inversions when a single particle travels along the axial direction. DH-PSF iSCAT microscopy was used to record the 3D trajectory of a single microbead attached to the flagellum, facilitating precise analysis of fluctuations in motor dynamics. Its ability to track single nanoparticles, such as 3D diffusion trajectories of 20 nm gold nanoparticles in glycerol solution, was also demonstrated. The DH-PSF iSCAT technique enabled by a 1D PC holds potential promise for future applications in physical, biological, and chemical science.

## Introduction

Single-particle tracking (SPT) is an immensely valuable technique used in microscopy to follow the motion of individual objects within a medium or in living cells, providing valuable information on their dynamic behavior over time, such as nanoscopic protein motion on a live cell membrane^1^. It can reveal the dynamics of individual particles or even molecules, and the motion of small objects can also serve as a probe for nanoscopic potentials^2,3^. If the particle is fluorescent, this technique is also referred to as single emitter tracking. There are a range of methodologies utilized in emitter localization and trajectory reconstruction, which achieve 2D and 3D subdiffraction tracking for wide applications in cell biology and other materials, such as localization microscopy by point-spread-function engineering^4,5^. The main advantage of fluorescence-based tracking is the low background intensity because the excitation wavelength is different from the emission wavelength, and the use of a bandpass filter can remove the background noise caused by the excitation beam. However, fluorescence-based tracking is limited by the photobleaching effect of fluorescent particles, and the intensity and duration of fluorescence emission impose fundamental limits on the imaging speed and precision for tracking studies^6^. Currently, much attention is being given to inventing brighter and more photostable tagging species that induce minimal perturbations to the analyte or host matrix^7^.

In recent decades, interferometric scattering (iSCAT) microscopy, which is a powerful label-free technique based on common-path interference between the light scattered from a particle of interest and a brighter reference field, usually reflected from (or transmitted through) the sample substrate, has developed rapidly^6,8–15^. Taking advantage of its intrinsic interferometric nature, iSCAT can track individual unlabeled particles with high spatial and temporal resolution, such as viruses, proteins, dielectric, or metallic nanoparticles^16–20^. Label-free 3D SPT using iSCAT microscopy is challenging due to multiple contrast inversions when this nanoparticle travels along the axial direction. The reason for the multiple contrast inversions is the different phase evolutions of the scattered light and the reference light^6,14^. During the contrast inversion process, this nanoparticle is hardly detected when its image contrast approaches zero, which is unfavorable for continuous axial tracking of this single particle. This challenge has been addressed through the adoption of ingenious software for postprocessing the captured iSCAT images^1,21,22^. However, in the general case of unknown particle size and refractive index parameters, the maximum contrast of the scattered signal of particles is uncertain, and the contrast of iSCAT PSF oscillates axially leading to multiple potential fitting solutions, increasing the complexity of PSF fitting during 3D particle positioning and tracking.

In this work, we address this challenge through the invention of hardware and a new effect, which is a well-designed all-dielectric one-dimensional photonic crystal (1D PC)^23,24^. This well-designed 1D PC can enhance the spin-to-orbital angular momentum conversion efficiency. When it is used as a substrate for iSCAT microscopy, it can efficiently transfer scattered light from a single particle to a point source with orbital angular momentum (OAM)^25,26^. The common path interference between scattered light with OAM and transmitted nonscattered illumination light (reference beam) results in an axial-location-dependent double-helix point spread function (DH-PSF) for iSCAT microscopy. DH-PSF will not experience multiple contrast inversions when a single particle travels along the axial direction, as occurs for iSCAT with a standard PSF, which will be more favorable for long-range and continuous 3D SPT. More importantly, the DH-PSF iSCAT image encodes the axial position of the single particle in the angular orientation of the DH-PSF lobes, which are distinctly above the background with approximately the same intensity throughout the entire axial range of interest. This approach provides a new means for 3D label-free SPT.

We applied this new iSCAT technique for 3D tracking of the rotation of the bacterial flagellum in a label-free manner. In studies of biological rotatory motors such as the F0F1 ATPase and the bacterial flagellar motor^27^, a microsphere with a diameter of approximately 500 nm is usually attached to the motor as an indicator of motor rotation. Here, we demonstrated the direct measurement of the 3D trajectory of bead rotation with high spatiotemporal resolution. The ability of the proposed iSCAT with DH-PSF to detect label-free single nanoparticles, such as gold nanoparticles with a diameter of approximately 20 nm and polystyrene nanoparticles with a diameter of 50 nm, was also successfully demonstrated over a long axial range. For example, the free diffusion trajectories of single 20 nm gold nanoparticles in glycerol solution were successfully measured. We successfully obtained the 3D diffusion trajectory of the nanoparticle and calculated its diffusion coefficient and hydrodynamic radius.

## Results

### Principle for the 1D PC enhancing spin-to-orbital angular momentum conversion

**Fig. 1:**
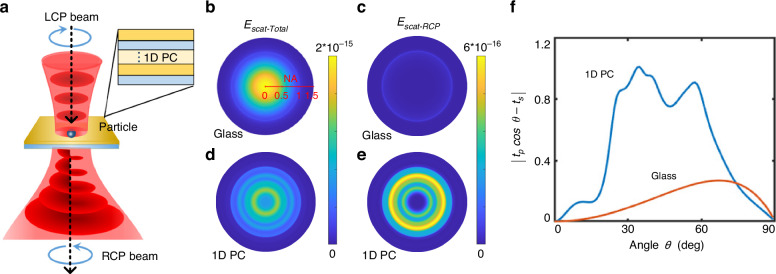
**The 1D PC enhances the spin-to-orbital angular momentum conversion of the scattered light from a single particle**. **a** Schematic of the spin-to-orbit angular momentum conversion for scattered light from a single particle under illumination by a circularly polarized beam. This particle can be placed on a glass or 1D PC substrate. The inset shows the structure of the 1D PC. Calculated far-field angular-dependent intensity distributions (BFP image) of the total scattered light (*E*_*scat-Total*_) from a single particle when the particle was placed on a glass (**b**) or 1D PC substrate (**d**), and the wavelength of the incident light was 635 nm. Calculated far-field angular-dependent intensity distributions (BFP image) of the RCP-scattered light (*E*_*scat-RCP*_) for the particle on the glass (**c**) or 1D PC substrate (**e**). **f** Calculated angular-dependent transmittance through the glass and 1D PC substrate, where *θ* is the incidence angle with respect to the direction normal to the substrate. NA = n*sin *θ*, where *n* is the refractive index

As shown in Fig. 1a, a single polystyrene particle (500 nm in diameter), the size of which is equivalent to a microscopic bead attached to the filament stub, is illuminated with a left-circularly polarized (LCP) and weakly focused (numerical aperture of the focusing objective is 0.13) laser beam. The incident wavelength is 635 nm. For comparison, the particles are placed on either a bare glass substrate or a dielectric 1D PC (inset of Fig. 1a). The all-dielectric 1D PC is made of alternating layers of Si_3_N_4_ and SiO_2_. There are 40 of these layers in total, and the thicknesses of the Si_3_N_4_ and SiO_2_ layers are 59 nm and 72 nm, respectively. Here, the 1D PC was designed for particles with a diameter of approximately 500 nm. Further details of the structural parameters of this 1D PC are provided in the Materials and Methods section.

Numerical simulations based on the finite difference time domain (FDTD) method are performed to calculate the far-field angular-dependent electric field distribution of the total forward scattered light (*E*_*scat-Total*_) and right-circularly polarized (RCP) forward scattered light (*E*_*scat-RCP*_) from a single particle on the substrate, as shown in Fig. 1b–e (corresponding to the back focal plane (BFP) images of the scattered light or the electric field distribution of the far-field scattered light in the Fourier space). Details on how to perform the numerical simulations are given in Section 1 of the Supplementary Information.

Because the single particle is illuminated by an LCP beam, the forward-scattered light from the particle on the glass substrate will be mainly LCP, as demonstrated in Fig. 1b, c, where the intensity of the scattered light in Fig. 1b is much stronger than that in Fig. 1c. However, due to the depolarization effects of light scattered from single particles, especially at large scattering angles (nonparaxial fields where a phenomenon known as spin–orbit coupling occurs^28–31^), the scattered light will also contain the RCP component (Fig. 1c) that is transferred from the LCP component during the scattering process. It should be noted that in our work, the light scattered by the single particles was collected by an oil-immersed objective of high numerical aperture (NA, 1.49); thus, the scattered light inside the objective (or inside the focal region) is composed of nonparaxial fields, and the spin-angular momentum (SAM) and OAM no longer separate from each other (a phenomenon known as spin-orbit coupling). However, when the scattered light is emitted out of the objective, it will be collimated and then focused by a low-NA tube lens to the camera. In these regions, the scattered light can be seen as paraxial fields (as shown in Fig. S2), and the SAM and OAM can be separated from each other.

The incident LCP beam carries a SAM of 1 ћ, and the collected RCP beam carries a SAM of −1 ћ. Then, based on the angular momentum conservation law, the RCP light collected by the camera that is transferred from the incident LCP light will carry an OAM with topological charge *l* = 2^26^. This means that the RCP light scattered from a single particle is a circularly polarized optical vortex beam, which is induced by the effect of spin-to-orbit angular momentum conversion^29–31^. However, the conversion efficiency of the particles placed on the glass substrate is much lower than that of the particles placed on the 1D PC, as shown in Fig. 1b–e, where the intensity of the RCP light scattered in Fig. 1c is much weaker than that in Fig. 1e. The 1D PC was designed to enhance the spin-to-orbit angular momentum conversion efficiency, where the single particle was placed on the 1D PC (Fig. 1a). This is the aim of the 1D PC design, which is realized through FDTD numerical simulations.

The principle of 1D PC substrate design can be theoretically analysed from the perspective of the transmission characteristics of circularly polarized light through two substrates, and more details on how the 1D PC structure enhances the spin-to-orbital angular momentum conversion of the transmission light are analysed in Section 2 of the Supplementary Information. In the following theoretical analysis, there was no particle placed on the 1D PC. The incident light beam is striking to the substrate at various angles$(\theta ,\varphi )$, and the angle-dependent transmittance $T(\theta ,\varphi )$ is derived as follows. On a circular coordinate basis, the incident beam is an LCP (defined as ${e}_{in}={(1,0,0)}^{T}$), and the output beam is an RCP (defined as ${e}_{out}={(0,1,0)}^{T}$); then, the transfer function $T(\theta ,\varphi )$ of the electric field collected by the imaging objective lens can be written as Eq. (1)^32,33^

1 $$T(\theta ,\varphi )={e}^{i2\varphi }\cdot \,\cos {\theta }^{-1/2}\cdot ({t}_{p}\,\cos \theta -{t}_{s})/2$$

Here, *θ* is the incidence angle with respect to the normal direction to the substrate, and *φ* is the azimuthal angle. The *t*_*p*_ and *t*_*s*_ are the electric field transmittances for the *p*- and *s*-polarized beams through either the glass or 1D PC substrate, respectively. The transfer function exhibits a nontrivial topological charge of two (*l* = 2)^34^. The transmittance curves of $|{t}_{p}\,\cos \theta -{t}_{s}|$ vs. angle *θ* for the two substrates are calculated, as shown in Fig. 1f, which shows that the curve for the well-designed 1D PC substrate is much greater than that for the glass substrate. A larger value of $|{t}_{p}\,\cos \theta -{t}_{s}|$ indicates a higher conversion efficiency from LCP light to RCP light, which was induced by the enhanced spin-to-orbital angular momentum conversion during the light transmission process through the designed 1D PC. The theoretical analysis verifies that the well-designed 1D PC can enhance the spin-orbit angular momentum conversion of the transmission light through this 1D PC.

A comparison of Fig. 1b, f shows that the angular distribution of the scattered light from the 500 nm particle approximately overlaps with the angular range of the curve (blue curve for 1D PC) with a large value of $|{t}_{p}\,\cos \theta -{t}_{s}|$. This means that this designed 1D PC substrate can efficiently convert LCP scattered light from 500 nm particles to RCP light containing OAM, and the designed structural parameters of the 1D PC are suitable for particles about this size. The angular distribution of the scattered light from a single particle does not change substantially when the particle size changes from 400 nm to 600 nm (Fig. S3a–c, the scattered light is mainly concentrated inside the critical angle), and the blue curve in Fig. 1f has a wide peak. Thus, this designed 1D PC substrate can work in a broad size range of single particles. On the other hand, when the particle is smaller than half the wavelength of the illumination light, the scattered light will have an obviously different angular distribution that is mainly concentrated at large angles (Fig. S3d–f, the scattered light is mainly concentrated outside of the critical angle); then, the 1D PC should be redesigned. More details on the design principle of a 1D PC and its dependence on the particle size are described in Section 3 of the Supplementary Information.

In experiments, the far-field angular-dependent intensity distributions through the two substrates can be measured with a back focal plane (BFP) imaging instrument, as shown in Fig. S4a. The measured BFP images (Fig. S4b–e) are consistent with the simulated images shown in Fig. 1b–e, which further verifies the enhanced spin-to-orbital angular momentum conversion induced by the properly designed 1D PC structure.

### The formation of a double-helix pattern based on enhanced spin-to-orbital angular momentum conversion

**Fig. 2:**
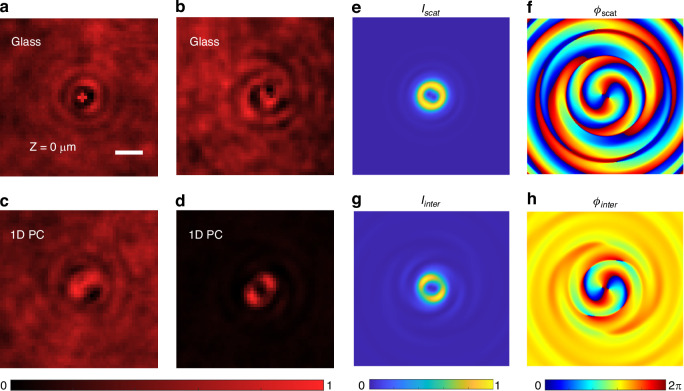
**Single-particle imaging via DH-PSF iSCAT microscopy**. DH-PSF iSCAT images for a particle on the glass substrate when the incident light and the detected light are of identical circular polarization (**a**) or nearly orthogonal polarization (**b**), (**c**, **d**) for a particle on the 1D PC substrate. **e**, **f** Simulated intensity (*I*_*scat*_) and spatial phase (*ϕ*_*scat*_) distributions of the forward-scattered light from a single particle. **g**, **h** Simulated intensity (*I*_*inter*_) and phase (*ϕ*_*inter*_) distributions of the interference field between the scattered light from the particle and the nonscattered transmitted light, which corresponds to the iSCAT image in (**d**). Scale bars in (**a**), 1 µm

The scattered light (signal field) from a single particle that contains the OAM can interfere with the transmitted nonscattered illumination light (reference field); thus, a new configuration of iSCAT microscopy was invented, as shown in Fig. S4a. The first combination (the first circular polarizer) of a quarter-wave plate (QWP1) and a polarizer (P1) is used to generate the LCP beam for illumination, and the second combination of QWP2 and P2 (the second circular polarizer) is used to select the RCP or LCP components of the light collected by the imaging objective (100×, numerical aperture NA 1.49), which is realized by tuning the orientation of the QWP2 and polarizer P2. When the particle was placed on the glass substrate, the captured images are shown in Fig. 2a, b, where the handedness of the illumination beam and collected beam were identical and nearly reversed, respectively. The image contrast did not obviously improve when the handedness of the illumination beam and collected beam was reversed when the particle was placed on the glass substrate. In contrast, when the particle is placed on the 1D PC substrate, the imaging contrast is highly enhanced when the illumination beam and the collected beam are reverse handed, as shown in the comparison between Fig. 2c, d, where the particle is much more obvious in Fig. 2d. In Fig. 2b, d, we slightly shifted the orientation of polarizer 2 (approximately 10°); then, a small portion of the transmitted nonscattered illumination light (reference field) can also reach the camera, which will interfere with the RCP-scattered light (carrying the OAM) from the single particle. This kind of interference results in a double-helix (DH) pattern on the image^4,35^.

The mechanism for imaging contrast enhancement can be analysed from the viewpoint of enhanced spin-to-orbit angular momentum conversion by the 1D PC. According to the calculation in Fig. 1f, the conversion efficiency of light at a small incident angle is low. The transmitted nonscattered light mainly has the same polarization as that of the illumination beam; thus, most of the transmitted light is blocked by the nearly orthogonal circular polarizer placed before the camera. The transmitted light intensity does not change much between the 1D PC and the glass substrate. On the other hand, there is a large angle of light in the scattered light of the particles, and the 1D PC significantly enhances the spin-orbit angular momentum conversion of the light at a large angle. Thus, compared to the particles placed in a glass substrate, the forward scattered light from the particle on the 1D PC will contain more RCP components. As a result, the imaging contrast of this particle is enhanced, as shown in Fig. 2b, d.

Due to the generation of a DH-PSF for iSCAT, a single particle with a diameter of 500 nm remains distinct when it is far from the focal plane of the imaging objective, as shown in Fig. S5a, b, where the distances between the particle and the focal plane are Z = −15 μm and Z = 15 μm, respectively. In contrast, when this particle was placed on the glass substrate, it appeared quite blurry in the images, as shown in Fig. S5c, d. More interestingly, the double spiral patterns of this particle (Fig. S5a, b) are reverse handed, which provides a new method to determine whether the particle is above or below the focal plane of the imaging objective.

To fully verify this ability, numerical simulations were performed to show the image formation of the proposed iSCAT microscope. The single particle is placed on the 1D PC substrate and is on the front focal plane (FFP) of the imaging objective. The orientation of polarizer P2 is slightly shifted (approximately 10°) so that some portions of the nonscattered illumination light can pass through polarizer 2 to reach the camera. Thus, interference between the scattered light from the particle and the transmitted nonscattered light can occur. Figure 2e shows the FFP electric field intensity distribution of the light scattered from the particle (*I*_*scat*_), and Fig. 2f shows the corresponding spatial phase distribution (*ϕ*_*scat*_) that verifies the generation of an optical vortex beam with topological charge *l* = 2. The interference between the scattered light and the transmitted light results in the pattern shown in Fig. 2g (*I*_*inter*_) and 2 h (*ϕ*_*inter*_). When the particle was far from the FFP of the objective, such as at *Z* = −15 μm and *Z* = 15 μm, the corresponding intensity distribution of the interference field could be simulated, as shown in Fig. S5e, f. The consistency between the experimental and simulated results verifies that the DH-PSF is formed by the interference between the optical vortex light scattered from the single particle and the background transmitted nonscattered light, which are of the same type of common-path interference used in the reported iSCAT. Thus, the proposed microscope can be named DH-PSF iSCAT, which has the high sensitivity and temporal resolution as the reported iSCAT due to the use of the same common-path interference.

### Axial localization of a single particle using the double-helix interference pattern

The pattern containing two lobes shown in Fig. 2d, g is similar to the DH-PSF of fluorescence microscopy, which was invented by W. E. Moerner’s group^4,35^ and is generated by modulating the fluorescence emission from single molecules with an external spatial light modulator (SLM) or phase mask. The advantages of the DH-PSF in enhancing the axial-tracking range of a single emitter over the standard PSF of a conventional lens have been successfully demonstrated in the literature. Here, DH-PSF was subjected to label-free iSCAT microscopy for the first time (DH-PSF iSCAT). It can be anticipated that the angular orientation of the two lobes in Fig. 2d, g can also be used for axial tracking of a single particle with high imaging contrast. To verify this function in the experiment, the sample containing the 1D PC substrate and the particle is placed on an XYZ Piezo sample stage that is used to precisely tune the axial position of the particle. Here, the imaging objective (100X, NA 1.49) is stable and fixed, and the stage moves along the z-axis.

**Fig. 3:**
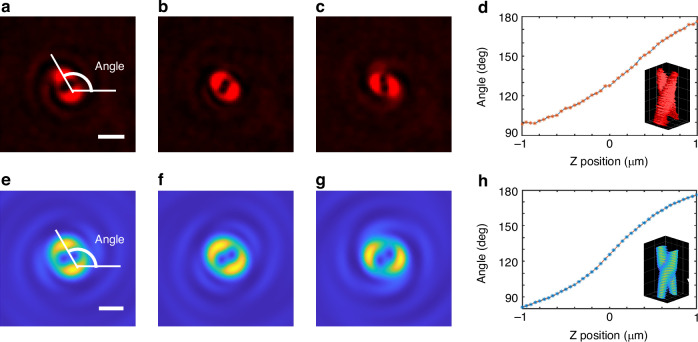
**Axial tracking of single particles via DH-PSF iSCAT microscopy**. **a**–**c** iSCAT images of the same particle at three different axial positions (Z-axis), (**d**) typical calibration curve of the angle between two lobes on (**a**–**c**) with respect to the axial position measured with a piezo-controlled stage. **e**–**h** Simulated DH-PSF iSCAT images and the corresponding calibration curve. Scale bars on (**a**, **e**), 1 µm

When the particle (polystyrene particle with a diameter of 500 nm) moves across the focal plane from top to bottom with a step of 50 nm, a series of images captured at each axial position (see Movies 1 and 2 in the Supplementary Information), such as the three images shown in Fig. 3a–c, are used to derive the calibration plot of the orientation angle versus axial motion, as shown in Fig. 3d. The plotted curve shows that the angular orientation of the two lobes changes with axial position, which is favorable for calibrating the axial position of a single particle.

The source MATLAB codes of the algorithm provided in ref. ^36^ are modified to process the captured images of DH-PSF iSCAT. The position of DH-PSF lobes was determined by using two different methods^4,37^: weighted centroid calculation and least-squares Gaussian fit. More details on how to derive the 3D position of the single particles are given in Section 4 in the Supplementary Information.

The average localization precision for 500 nm polystyrene particles within the entire axial working range is $({\sigma }_{x},\,{\sigma }_{y},\,{\sigma }_{z})\,=\,(1.4\,nm,\,1.6\,nm,\,8.2nm)$, which is beyond the diffraction limit. More detailed descriptions of the localization precision of the DH-PSF iSCAT method are provided in Section 5 and Fig. S7 of the Supplementary Information, which also includes a discussion of the influence of aberrations on axial localization.

The simulation results shown in Fig. 3e–h are consistent with the experimental results, thus verifying that the new iSCAT microscope can be used to track the motion of a single particle over an axial range of 2 μm. This axial range of 2 μm is larger than the DOF of the imaging objective (the DOF is twice the Rayleigh distance ${{\rm{Z}}}_{R}=\frac{\pi {\omega }_{0}^{2}}{\lambda }$, where ${\omega }_{0}$ is the radius of the focusing spot and *λ* is the wavelength, which is approximately 500 nm), which is the typical advantage of DH-PSF microscopy^4,35^. The double-helix fluorescence PSF was used for single-emitter tracking over an axial range of 2 μm. It is a fluorescence-based tracking method, and an external SLM or phase mask is needed to realize the double helix PSF. Here, DH-PSF was introduced to label-free iSCAT microscopy, which shares the same advantages as the iSCAT technique, such as a simple and robust configuration and high sensitivity to nano-objects due to the use of common-path interference. Notably, there are no multiple contrast inversions when this single particle travels along the axial direction, as shown in Movies S1 and S2. This means that the challenge of label-free 3D SPT using iSCAT microscopy can be addressed through the adoption of the effect of a 1D PC enhancing spin-to-orbital angular momentum conversion.

### Theoretical mechanism for the axial localization of a single particle using a DH-PSF

The mechanism for axial tracking enabled by DH-PSF iSCAT microscopy can be briefly described as follows, and detailed analyses are given in Section 6 of the Supplementary Information. The interference field between the scattered light field (point source of optical vortex with topological charge *l* = 2, which can be approximately seen as a Laguerre Gaussian beam^38–40^,*E*_*s*_) and the transmitted light field *E*_*t*_ can be written as Eq. (2):

2 $$I={|{E}_{s}+{E}_{t}|}^{2}={|{E}_{s}|}^{2}+{|{E}_{t}|}^{2}+2|{E}_{s}||{E}_{t}|\cos \Delta \phi$$

Here, Δ*ϕ* is the phase difference between the two fields and can be written as Eq. (3)^41^:

3 $$\Delta \phi =2\varphi +k\frac{{r}^{2}}{2{R}_{s}(z)}-3\arctan \frac{z}{{z^{\prime} }_{0}}$$

where 2*φ* is the second-order phase vortex, ${R}_{s}(z)$ is the spherical wavefront, $\arctan \frac{z}{{z{\prime} }_{0}}$ is the Gouy phase and ${z{\prime} }_{0}$ is the Rayleigh range of the Laguerre Gaussian beam. Based on Eq. (3), the experimental and simulation phenomena shown in Figs. 2 and 3 can be analysed as follows.

In the vicinity of the focus, as the axial position of the particle changes, the Gouy phase shifts. Thus, the two lobes generated by interference between scattered light and transmitted light rotate around the optical axis (Z-axis). In areas far from the focus (such as Z = 15 μm or Z = −15 μm), the spherical wavefronts of the scattered light above and below the focal plane are opposite. As a result, the rotation directions of the spiral pattern handedness are reversed (as shown in Fig. S5).

### 3D tracking of a microbead attached to the filament stub

**Fig. 4:**
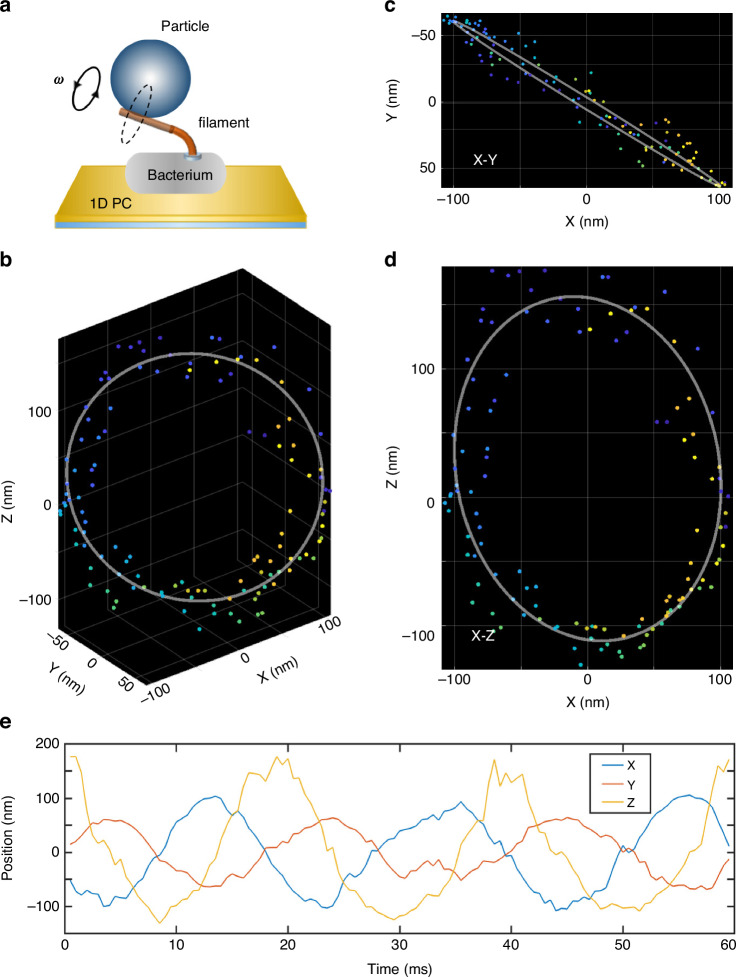
**3D motion tracking of the particle attached to the filament stub of a living bacterium**. **a** Schematic view of the sample. A living bacterium was adhered to the 1D PC substrate with a particle (500 nm in diameter) attached to the filament stub that rotated on an approximately circular trajectory. **b** 3D trajectories of the particle observed by DH-PSF iSCAT microscopy. **c**, **d** The projected trajectories on the X-Y and X-Z planes. **e** Graph of the 3D spatial position (X, Y, Z) of a particle changing over time

Here, a living bacterium is adhered to the 1D PC, and a 500-nm-diameter latex bead is attached to the shortened filament stub (Fig. 4a). The bacteria were glued to the substrate with 0.1% poly-L-lysine, which is a commonly used reagent in biology. The bead rotates on an approximately circular trajectory. Only the 2D projection of the bead trajectory was recorded previously, and the corresponding 3D trajectory was reconstructed by assuming a circular trajectory and projecting the 2D ellipse back to a circle^42,43^. During this reconstruction process, spatial resolution worsened, and important details of motor dynamics were lost. Here, we recorded the 3D trajectory of bead rotation with DH-PSF iSCAT microscopy at 2000 fps, as shown in Fig. 4b. The 2D projections of the bead trajectory on the X-Y and X-Z planes are shown in Fig. 4c, d, respectively. A graph of the change in the 3D spatial position of the bead over time is also shown in Fig. 4e. Due to the marker-free imaging method, the temporal resolution is much greater than that of methods using fluorescence; thus, the time needed for one revolution of the 3D trajectory is approximately 19 ms. The experimental results clearly verify that the bead truly rotates with a radius of approximately 110 nm in 3D space. Details of the formations of the 3D trajectory are shown in Movie 3 in the Supplementary Information.

### 3D single-nanoparticle tracking with DH-PSF iSCAT microscopy

The above 1D PC substrate is designed for particles with a diameter of approximately 500 nm, which is typically used in the study of bacterial flagellar motor dynamics under high loads. By properly tuning its structural parameters, a 1D PC can be designed to enhance the spin-to-orbit angular momentum conversion of scattered light from smaller nanoparticles with diameters less than 100 nm, such as gold nanoparticles with diameters of approximately 20 nm and polystyrene nanoparticles with diameters of approximately 50 nm. It is well known that the scattering characteristics of a single nanoparticle with a diameter much smaller than the incident wavelength can be approximated by an electric dipole; thus, the 1D PC substrate can be optimized based on the radiation field of an electric dipole, as shown in Fig. S9. Based on the numerical simulations, the scattering angles of the light from a tiny nanoparticle (or electric dipole) are always larger than those from larger single particles, as shown in the comparisons between Figs. 1b and S9a^44^. According to this characteristic, we optimized the structural parameters of the 1D PC substrate for small nanoparticles. Here, the thickness of the SiO_2_ layer is 157 nm, and that of the Si_3_N_4_ layer is 46 nm. There are 20 of these layers in total.

**Fig. 5:**
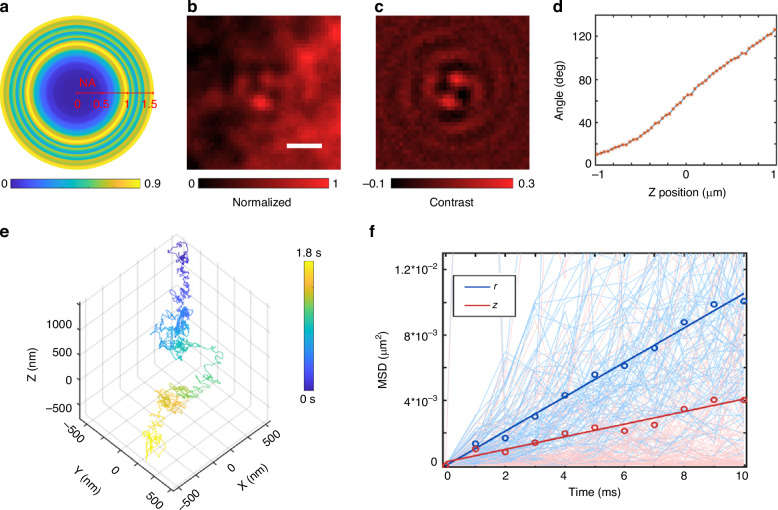
**3D single nanoparticle tracking with DH-PSF iSCAT microscopy**. **a** The simulated image shows the ratio of the angular-dependent electric field intensity between the RCP radiation light and the total radiation light from an electric dipole that is placed on a 1D PC. **b** Raw DH-PSF iSCAT image of a single 20 nm Au nanoparticle. **c** Resulting ratiometric image of a 20 nm Au particle obtained by subtracting the background without particles. **d** The calibration curve of the angular orientation vs. axial position of the Au nanoparticle. **e** Representative trajectory of a single 20 nm Au nanoparticle in glycerol solution with a duration of 1.8 s and an exposure time of 1 ms. **f** Plots of the mean-square displacement (MSD) in *r* and *z* versus diffusion time (blue for *r*, pink for *z*) for a nanoparticle’s trajectory over 1 s. The thin lines show the MSD extracted from each individual trajectory. The thick circles and lines show the weighted average and a linear fit to the MSD for displacements *r* and *z*, respectively

Similar to what is shown in Fig. 1, when an electric dipole is placed on the 1D PC substrate, the angular-dependent intensity ratio between the forward RCP-radiation field and the total radiation field of the electric dipole was calculated, as shown in Fig. 5a. This demonstrates that a higher spin-to-orbit angular conversion efficiency occurs at a larger scattering angle (or larger NA = n*sin *θ*, where *θ* denotes the scattering angle), verifying that the new 1D PC is suitable for smaller nanoparticles.

In the experiments, 20 nm gold nanoparticles were placed on this new 1D PC substrate, and scanning electron microscopy (SEM) images of the nanoparticles are shown in Fig. S10 of the Supplementary Information. The sample containing the new 1D PC substrate and the 20 nm gold nanoparticle is placed on an XYZ Piezo sample stage that is used to precisely tune the axial position of this nanoparticle. Here, the imaging objective (100X, NA 1.49) is stable and fixed, and the stage moves along the Z-axis, similar to the setup shown in Fig. S4a. The captured image (Fig. 5b) of the single gold nanoparticle shows two lobes that can be more distinct when background subtraction is conducted, as shown in Fig. 5c. Here, the definition of image contrast is based on the reference^45^, which is always used for small nanoparticles imaged by iSCAT (see the Materials and methods section).

When the 20 nm gold nanoparticle moved across the focal plane from top to bottom with a step of 50 nm, a series of images captured at each axial position were used to derive the calibration plot of the orientation angle versus axial motion, as shown in Fig. 5d. By recording 100 images of this gold nanoparticle when it was nearly on the focal plane, we obtained the standard deviation of the nanoparticle position $({\sigma }_{x},\,{\sigma }_{y},\,{\sigma }_{z})\,=\,(7.1\,nm,\,7.6\,nm,\,12.3\,nm)$, which shows the subdiffraction-limited localization ability. DH-PSF iSCAT microscopy images of single polystyrene nanoparticles with diameters of 50 nm and 100 nm on the same 1D PC are also shown in Fig. S11. These experimental results verify the ability of this DH-PSF iSCAT microscope for tracking single nanoparticles over a long axial range.

### 3D diffusion trajectories of single gold nanoparticles recorded by DH-PSF iSCAT

Here, we demonstrate how to track and analyse the free diffusion of single gold nanoparticles (with a diameter of 20 nm) in a glycerol solution. Single nanoparticles were diluted in aqueous glycerol to a volume fraction of 85%. Figure 5e shows a representative trajectory of a single gold nanoparticle in aqueous glycerol with an exposure time of 1 ms (see Movie 4 in the Supplementary Information). The trajectory consists of 1800 frames, which is equivalent to a total trajectory time of 1.8 s. By locating the *x*, *y*, and *z* positions of the nanoparticle, as well as the diffusion time Δ*t*, the mean square displacement (MSD) of the nanoparticle can be obtained. The MSD is defined by

4 $${\rm{MSD}}({\Delta}t)={\langle|{r}(t+\Delta{t})-r(t)|\rangle}^{2}$$

It can be assumed that the motion of the nanoparticle is pure diffusion. The MSD scaling versus $\Delta t$ is given by

5 $${\text{MSD}}(\Delta t)=2nD\Delta t+\mathop{\sum }\limits_{i}^{n}2{\sigma }_{i}^{2}$$

where *n* is the number of dimensions, *D* is the diffusion coefficient, and *σ*_*i*_ is the position uncertainty of each dimension^35^. A plot of the MSD in *r* and *z* of a nanoparticle’s trajectory over 1 s vs. diffusion time $\Delta t$ is shown in Fig. 5f. The trend of the MSD with respect to *r* and *z* is linear, and the MSD of *r* is approximately 2.8 times that of *z*, which is also consistent with the behavior of 3D free diffusion $({\text{MSD}}(r)=3{\text{MSD}}(z))$. The diffusion coefficient *D* extracted from the fit is $0.18\,{{\mu }}{{\rm{m}}}^{2}{{\rm{s}}}^{-1}$, and the viscosity *η* of the glycerol solution at *T* (295 K) is approximately $0.1\,{\rm{Pa}}\cdot {\rm{s}}$. According to the Stokes–Einstein (SE) equation,

6 $$D=\frac{{k}_{{\rm{B}}}T}{3\pi \eta d}$$

where *k*_*B*_ is the Boltzmann constant and *d* is the diameter of the particle. The calculated hydrodynamic diameter of the nanoparticles is 24 nm, and the larger calculated diameter value than the real diameter (approximately 20 nm) may be due to the higher actual viscosity of glycerol than estimated. It should be noted that the proposed method can also record fast nanoparticle movements if a camera with high frames per second (fps) is used to record the images. The measured trajectory of a single gold nanoparticle in aqueous glycerol successfully proves that DH-PSF iSCAT can track the 3D motion of single nanoscale particles.

### Comparisons between the standard PSF and DH-PSF images

**Fig. 6:**
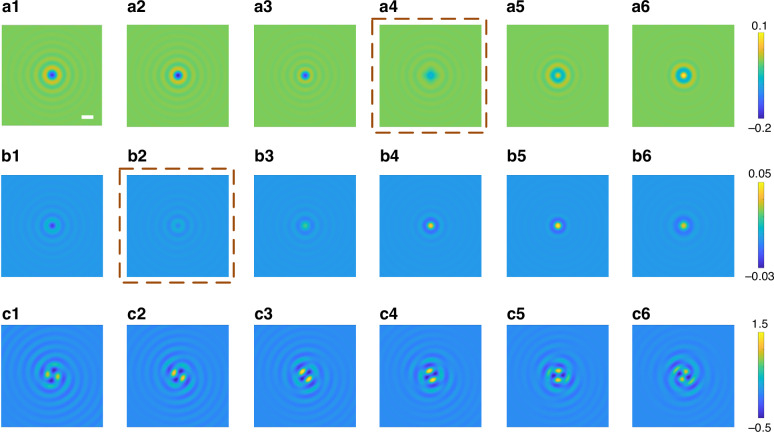
**Simulated PSF images of standard iSCAT microscopy and DH-PSF iSCAT microscopy**. **a1**–**a6** Standard PSF image of iSCAT working in reflection mode; (**b1**–**b6**) standard PSF image of iSCAT working in transmission mode (also named COBRI); (**c1**–**c6**) DH-PSF iSCAT image. The imaged object is a single polystyrene particle with a diameter of 100 nm. The three rows of images are recorded when this single nanoparticle moves axially within a range of 1 μm near the DOF of the imaging objective lens (100 X, NA 1.49). The axial distance traveled by the nanoparticle on adjacent images is 200 nm. Scale bar, 1 µm. The right color bars denote the imaging contrast, whose definitions are given in the Materials and methods section

Finally, we directly demonstrate the differences between the standard PSF and DH-PSF through numerical simulations, where a single nanoparticle (polystyrene particle with a diameter of 100 nm) travels along the axial direction. The simulated standard PSF (Fig. 6a, b) of iSCAT working in the reflection mode and transmission mode (also named COBRI) shows that the ring pattern and contrast of the single nanoparticle on the standard PSF images change with the axial location of this single nanoparticle. For a standard PSF, the axial location of the particle is always encoded by the diameter of the rings and the central contrast. When a single particle moves axially (within a range of 1 μm near the DOF region of the imaging objective), the standard PSF images of iSCAT and COBRI undergo contrast reversal (the center of the interference pattern changes from dark to bright) due to the different phase evolutions of the scattered light and the reference light. When this nanoparticle is located at some axial locations, its image contrast is nearly zero (such as that shown in Fig. 6-a4, b2), and it will be difficult to find this single nanoparticle, thus affecting the continuous tracking of the particle. In addition, the local optimization method is usually used to fit the iSCAT PSF and the connected particle position. Given the limited axial motion between frames, the fitted output of the previous frame can be carried into the parameter initialization of the next frame. However, in the general case of unknown particle size and refractive index parameters, the maximum contrast of the particle scattered signal is uncertain. Moreover, the contrast of iSCAT PSF oscillates with the axial position and there are multiple potential fitting solutions, which increases the complexity of PSF fitting in the process of 3D particle localization and tracking.

On the other hand, as shown in Fig. 6c, the DH-PSF iSCAT image maintains nearly the same contrast, where the two lobes are distinctly above the background with approximately the same contrast. The axial location of this nanoparticle is encoded in the angular orientation of the two lobes, which rotates continuously as the nanoparticle continuously travels along the axial direction. The information theory analysis shows that compared to the traditional PSF, the DH-PSF provides more uniform Fisher information for 3D position estimation. The image contrast of the DH-PSF image is also larger than that of the standard PSF image, as indicated by the color bar in Fig. 6. Comparisons of the three rows of simulated PSF images demonstrate that DH-PSF will be more favorable for long-range and continuous axial tracking of a single nanoparticle.

## Discussion

In summary, we have proposed a new configuration for label-free iSCAT microscopy that involves DH-PSF through the adoption of a well-designed 1D PC. The DH-PSF design features high and uniform Fisher information and has two dominant lobes in the image plane. The DH-PSF lobes are distinctly above the background with approximately the same intensity through the axial range of nearly 2 μm for a 100X objective, which is approximately four times the DOF of this objective lens. The angular orientation of the two lobes rotates with the axial position of the single particles, thus providing a new route for tracking single particles over a long axial range, such as tracking single polystyrene particles with diameters of 500 nm, 100 nm, or 50 nm and gold nanoparticles with a diameter of 20 nm. Thus, DH-PSF iSCAT microscopy combines the advantages of both the DH-PSF and iSCAT techniques, including high sensitivity enabled by common-path interference, enhanced image contrast, and a long axial tracking range (approximately 2 μm) enabled by DH-PSF.

The working principle relies on the adoption of a 1D PC that enhances the spin-to-orbit angular momentum conversion effect to the iSCAT technique, which improves both the imaging contrast and axial-tracking range of iSCAT microscopy. OAM and spin-to-orbit angular momentum conversion have been hot topics in the optics and photonics community over the past 30 years^46–48^, and now, they are being introduced to iSCAT for the first time, which will expand the functions of the iSCAT technique and make it more attractive and interesting to researchers in optics and photonics. In the future, combined with the ingenious data/image postprocessing methods and PSF engineering methods developed by the pioneers of iSCAT and PSF engineering microscopy^49–52^, the proposed DH-PSF iSCAT will be more powerful and provide new opportunities for future applications in biological, chemical and physical science^49,50^.

## Materials and methods

### Fabrication of the 1D PC

The all-dielectric 1D PC was fabricated via plasma-enhanced chemical vapor deposition (PECVD, Oxford System 100, UK) of SiO_2_ and Si_3_N_4_ layers on a microscope cover slip (thickness: 0.17 mm) at a vacuum of <0.1 mTorr and a temperature of 300 °C. Before the deposition process, the coverslip was sequentially cleaned using acetone, absolute ethanol, piranha solution, and nanopure deionized water and then dried using a N_2_ stream. The process of Si_3_N_4_ generation is dependent on the chemical reaction of SiH_4_ with N_2_O and NH_3_ at high temperatures, and the process of SiO_2_ generation is dependent on the chemical reaction of SiH_4_ and N_2_O. The refractive index of SiN_x_ can be adjusted by changing the ratio of SiH_4_ to NH_3_. By controlling the ventilation volumes and ventilation rates of the various gases, the thickness and refractive index of each layer can be determined precisely. The SiO_2_ layer is a low refractive index dielectric layer (n = 1.46), and the Si_3_N_4_ layer is a high refractive index dielectric layer (n = 2.53 or n = 2.14). Nanoparticles (such as polystyrene nanoparticles or Au nanoparticles with diameters of 20 nm, 50 nm, and 100 nm) were placed on a well-designed dielectric 1D PC substrate (alternative layer of silicon nitride (Si_3_N_4_, refractive index n = 2.14, 46 nm thickness) and layer of silicon dioxide (SiO_2_, n = 1.46, 157 nm thickness)). There are 10 pairs of Si_3_N_4_ layers + SiO_2_ layers. For polystyrene microparticles (such as single polystyrene particles with a diameter of 500 nm), the 1D PC substrate is composed of an alternative layer of silicon nitride (Si_3_N_4_, refractive index n = 2.53, 59 nm thickness) and a layer of silicon dioxide (SiO_2_, n = 1.46, 72 nm thickness). There are 20 pairs of Si_3_N_4_ layers + SiO_2_ layers. This 1D PC structure does not require precise nanofabrication procedures on the surface and can be manufactured on a large scale at low cost with standard deposition methods. The cost for each piece of the 1D PC substrate is only tens of yuan (RMB)^53^. We provide a photo of the fabricated all-dielectric 1D PC as shown in Fig. S12 in the Supplementary Information.

### Preparation of single particles and biological samples

The polystyrene particles were purchased from Thermo Fisher Scientific (USA). The certified mean diameter of the particles supplied was approximately 500 nm. The particles dispersed in either water or ethanol solution were placed on the substrates. If single particles are dispersed in an ethanol solution, the particles will be exposed to air because the alcohol is volatile. The bacterial strain we used was JY27, which was derived from the *E. coli* K12 strain RP437 with motors rotating exclusively counterclockwise. The plasmid pKAF131 constitutively expresses sticky FliC. The cells were grown at 33 °C under vigorous shaking (200 rpm) in 10 mL of T-broth (1% [w/v] tryptone and 0.5% [w/v] NaCl) supplemented with 50 μg mL^−1^ chloramphenicol to an OD600 (optical density at 600 nm) between 0.45 and 0.50, and 1 ml of the grown culture was harvested by centrifugation at 4000 × *g* for 2 min, washed twice with 1 ml of motility medium [10 mM potassium phosphate, 0.1 mM EDTA, 1 μM methionine, and 10 mM lactic acid, pH 7.0], and resuspended in 1 ml of this medium. We truncated the flagella by passing 1 ml of the washed cell suspension between two syringes equipped with 23-gauge needles connected by 7-cm-long polyethylene tubing (0.58 mm inside diameter, no. 427411; Becton Dickinson) 120 times. The sheared cell suspension was centrifuged and resuspended in 0.3 ml of motility medium. The sheared cells were immobilized on a coverslip coated with poly-L-lysine (0.01%, P4707; Sigma, St. Louis, MO) assembled in a flow chamber and allowed to stand for 6 min. Then, 0.5-μm-diameter polystyrene beads (0.27%, Polysciences) were used to replace the suspension and attached to the sheared filament stubs by allowing it to stand for 6 min.

### Optical experiments

All optical measurements were performed using a custom-built optical microscope in transmission mode (Fig. S4a). The illumination beam is emitted from a laser diode with a wavelength of 635 nm. In this work, we achieve DH-PSF by designing transmittance functions instead of using the resonance mechanisms of a 1D PC, and this effect can be achieved in a certain range of wavelengths but is not fixed at a single resonance wavelength. Conventional iSCAT microscopy always involves a laser with a fixed wavelength working as the illumination source, so the 1D PC is well designed according to this wavelength. If the laser source was changed, we could redesign the structure of the 1D PC.

In our experiments, the incident power for illumination was approximately 0.04 kW cm^−2,^ and the image acquisition rate was 1000 fps for polystyrene microparticles (diameter of 500 nm). For smaller nanoparticles (such as 20 nm Au nanoparticles and 50 nm/100 nm polystyrene nanoparticles), the incident power for illumination is approximately 20 kW cm^−2^. The beam was collimated and passed through polarizer 1 and quarter waveplate 1. By properly tuning the orientations of the two elements, a left or right circularly polarized beam can be generated. In our experiments, the LCP beam was used for illumination, which was then focused onto the sample using objective 1 (UPlanFLN, 4×, NA = 0.13; Olympus, Japan). The samples consisted of particles or live bacteria placed on glass or 1D PC substrates. The sample was placed on an XYZ Piezo sample stage (model P-545.3C8S, Physik Instrumente, Germany); thus, the lateral and axial positions of the particles attached to the coverslip could be precisely controlled. The signals transmitted through the samples were collected using objective 2 (CFI Apochromat TIRF, 100×, NA = 1.49; Nikon, Japan). Another combination of quarter waveplate 2 and polarizer 2 is used to filter the transmitted signals so that only RCP signals can reach the detector (Prime 95B Scientific CMOS camera, TELEDYNE PHOTOMETRICS, Canada). By properly tuning the orientation of polarizer 2, both the forward scattered light from a single particle and the transmitted nonscattered light can reach the detector, thus resulting in a double-helix interference pattern.

For a single 500 nm polymer particle, the scattering intensity on the DH-PSF iSCAT microscope is much greater than the image background intensity; thus, there is no need to adopt background subtraction, so we define the image contrast as the contrast as the difference between the maximum and minimum image intensity values divided by their sum. This definition is the same as that used in reference^54^, which is suitable for images of large specimens.

However, for small nanoparticles (such as 20 nm gold nanoparticles and 50 nm/100 nm polystyrene nanoparticles), another definition of image contrast should be used. Based on the literature^45^, iSCAT/COBRI microscopy measures the interference between the scattered signal and the nonscattered transmitted/reflected reference beam. The detected intensity can be written as

7 $${I}_{{\rm{det}}}={|{E}_{{\rm{ref}}}|}^{2}+{|{E}_{{\rm{sca}}}|}^{2}+2|{E}_{{\rm{ref}}}||{E}_{{\rm{sea}}}|\cos \varphi$$

where ${I}_{{\rm{ref}}}={|{E}_{{\rm{ref}}}|}^{2}$ and ${I}_{{\rm{sca}}}={|{E}_{{\rm{sca}}}|}^{2}$ denote the intensities of the reference and the scattered light, respectively. The phase *φ* represents the relative phase between the two fields, which can arise from a Gouy phase in the imaging system. Then, the imaging contrast can be defined as

8 $${\rm{contrast}}=\frac{{I}_{{\rm{det }}}-{I}_{{\rm{ref}}}}{{I}_{{\rm{ref}}}}=\frac{{|{E}_{{\rm{sca}}}|}^{2}+2|{E}_{{\rm{ref}}}||{E}_{{\rm{sca}}}|}{{|{E}_{{\rm{ref}}}|}^{2}}$$

In our work, the image contrast for 20 nm gold nanoparticles (50 nm/100 nm polystyrene nanoparticles) was derived from the above definition, as shown in Figs. 5c, 6 and S11.

## Supplementary information

Supplementary Information for One-dimensional photonic crystal enhancing spin-to-orbital angular momentum conversion for single-particle tracking

Movie 1

Movie 2

Movie 3

Movie 4

## Data Availability

All study data are included in this article and/or the SI Appendix.
