# Supplementary material for: One-dimensional photonic crystal enhancing spin-to-orbital angular momentum conversion for single-particle tracking
Source: Light Sci Appl. 2024 Sep 26;13:268. doi: 10.1038/s41377-024-01623-x (PMC11427464; doi:10.1038/s41377-024-01623-x)
Supplement: Supplementary file 1 — Supplementary Information for One-dimensional photonic crystal enhancing spin-to-orbital angular momentum conversion for single-particle tracking [file 41377_2024_1623_MOESM1_ESM.docx]

**Supplementary Information for**

One-dimensional photonic crystal enhancing spin-to-orbital angular momentum conversion for single-particle tracking

Mingchuan Huang^1^†, Qiankun Chen^1^†, Yang Liu^1^, Chi Zhang^2^, Rongjin Zhang^2^, Junhua Yuan^2^*, and Douguo Zhang^1,3,4^*

^1^Advanced Laser Technology Laboratory of Anhui Province, Department of Optics and Optical Engineering, University of Science and Technology of China, Hefei, Anhui, 230026, China

^2^Department of Physics, University of Science and Technology of China, Hefei, Anhui, 230026, China

^3^Hefei National Research Center for Physical Sciences at the Microscale, University of Science and Technology of China, Hefei, Anhui 230026, China.

^4^Hefei National Laboratory, University of Science and Technology of China, Hefei 230088, China.

†Mingchuan Huang and Qiankun Chen contributed equally to this work.

*Correspondence and requests for materials should be addressed to [dgzhang@ustc.edu.cn](mailto:dgzhang@ustc.edu.cn) (Douguo Zhang) or [jhyuan@ustc.edu.cn](mailto:jhyuan@ustc.edu.cn) (Junhua Yuan).

**Section 1: Numerical calculations of the iSCAT microscopy image**

The calculation procedure for the iSCAT microscopy image can be divided into two steps. In the first step, the near-field electromagnetic field distribution is obtained via the finite difference time domain (FDTD) method. In the second step, the electric field at the back focal plane (BFP) is obtained through a near-to-far-field transformation ^1^. The iSCAT image (front focal plane image, FFP) is obtained by performing a two-dimensional Fourier transformation of the previous BFP image, considering the phase delay and polarization selection that are induced by the quarter waveplate and the polarizer.

**Step 1: The simulation of the near-field distribution**$E_{n}$

As shown in Figure S1, perfectly matched layer (PML) absorbing boundaries are used in three dimensions, and the boundaries form a simulation area with a size of 110 μm × 110 μm × 5 μm. Two light sources are used in the simulation. One is a Gaussian beam polarized along the X-axis and is focused by a 0.13 NA lens. The dimensions of this light source are set to 110 μm × 110 μm at a height of Z = 1.2 μm. As illustrated in Figure S1, the beam wavelength was set at 635 nm, and the surrounding medium was a vacuum. The other light source is also a Gaussian beam with a polarization orientation along the Y-axis, and its phase is 90° ahead of the first source. The two linearly polarized light sources are combined to simulate circularly polarized light.

Nanoparticles (such as polystyrene nanoparticles or Au nanoparticles with diameters of 20 nm, 50 nm, and 100 nm) were placed on a well-designed dielectric one-dimensional photonic crystal (1D PC) substrate (alternate layer of silicon nitride Si_3_N_4_, refractive index n = 2.14, 46 nm thickness and layer of silicon dioxide SiO_2_, n = 1.46, 157 nm thickness). There are 10 pairs of Si_3_N_4_ layers + SiO_2_ layers.

For polystyrene microparticles (such as single polystyrene particles with a diameter of 500 nm), the 1D PC substrate is composed of an alternate layer of silicon nitride (Si_3_N_4_, refractive index n = 2.53, 59 nm thickness) and a layer of silicon dioxide (SiO_2_, n = 1.46, 72 nm thickness). There are 20 pairs of Si_3_N_4_ layers + SiO_2_ layers. The structural dimensions of the dielectric 1D PC were the same as those used in the experiments. The monitor is placed in glass (n = 1.52), whose size is 110 μm × 110 μm, and is located at Z = -3 μm. This monitor can obtain the near-field electric field distribution $E_{n}$ of the iSCAT signal.

**Step 2:** **Calculation of the electric field distribution at the BFP** $E'_{b}$ **and FFP** $E_{i}$**.**

As shown in Figure S2, the calculation of the far field $E_{f}$ can be derived through a near-to-far-field transformation. According to the vector ray tracing ^2, 3^, the electric field at the BFP of the imaging objective lens $E_{b}$ is expressed as

|  |  | (**S1.1**) |
| --- | --- | --- |
|  |  | (**S1.2**) |

where is the transformation matrix, matrix describes the coordinate transformation for rotation around the Z-axis and (azimuthal angle) is the angle of the meridional plane to the X-axis.

|  |  | (**S1.3**) |
| --- | --- | --- |

where the matrix $L(\theta)$ describes the refraction of the ray as it traverses a lens and $\theta$ (incident angle) is the angle at which the ray rotates in a direction perpendicular to the meridional plane:

|  | $L(\theta)=\sqrt{\cos\theta}\cdot\left(\begin{matrix} \cos\theta& 0 & \sin\theta\\ 0 & 1 & 0 \\ -\sin\theta& 0 & \cos\theta\end{matrix}\right)$ | (**S1.4**) |
| --- | --- | --- |

The $E_{b}$ can be decomposed into two components, $E_{bx}$ in the X-direction and $E_{by}$ in the Y-direction, and $E_{by}$ is then superimposed with a phase delay of 90° to simulate the function of the quarter wave plate (QWP) used in the experiment. After an analyser is set to an angle $\alpha$ with respect to the X-axis, the electric field $E'_{b}$ can be written as:

|  | $\begin{matrix} E'_{bx}=\left[E_{bx}\left(cos\alpha\right)+E_{by}\mathrm{e}^{\frac{i\pi}{2}}\left(sin\alpha\right)\right]cos\alpha\\ E'_{by}=\left[E_{bx}\left(cos\alpha\right)+E_{by}\mathrm{e}^{\frac{i\pi}{2}}\left(sin\alpha\right)\right]sin\alpha\end{matrix}$ | (**S1.5**) |
| --- | --- | --- |

Finally, the electric field $E_{i}$ at the image plane is obtained through a two-dimensional Fourier transformation

|  | $E_{i}(x,y)=\iint E'_{b}(k_{x},k_{y})\exp(ik_{x}x+ik_{y}y)dk_{x}dk_{y}$ | (**S1.6**) |
| --- | --- | --- |

where $(x,y)$ is the lateral coordinate on the imaging plane of the tube lens and $(k_{x},k_{y})$represents the lateral coordinate on the momentum space.

**Section 2: Theoretical analysis of the spin-to-orbital angular momentum conversion of transmitted light through a dielectric 1D PC**

For an optical imaging system that uses vector ray tracing, the light scattered by a single particle passes through a 1D PC or a glass substrate and is collected by a high numerical aperture (NA) objective lens. On a circular coordinate basis ^4^, the transformation matrix of the electric field takes the form

|  |  | (**S2.1**) |
| --- | --- | --- |
|  |  | (**S2.2**) |

The angle- and polarization-dependent transmittance through a glass or 1D PC substrate is described by the matrix $T(\theta)$:

|  | $T(\theta)=\left(\begin{matrix} t_{p}(\theta) & 0 & 0 \\ 0 & t_{s}(\theta) & 0 \\ 0 & 0 & t_{p}(\theta) \end{matrix}\right)$ | (**S2.3**) |
| --- | --- | --- |

The matrix $V$ is a unitary transformation from a Cartesian to a circular basis:

| $V=\frac{1}{\sqrt{2}}\left(\begin{matrix} 1 & 1 & 0 \\ i & -i & 0 \\ 0 & 0 & \sqrt{2} \end{matrix}\right)$ | (**S2.4**) |
| --- | --- |

In our work, $e_{in}=(1,0,0)^{T}$ is left circularly polarized (LCP, incident beam), and $e_{out}=(0,1,0)^{T}$ is right circularly polarized (RCP, output beam); then, the transfer function can be given as follows:

|  |  | (**S2.5**) |
| --- | --- | --- |

According to Eq. (S2.5), the transfer function exhibits a nontrivial topological charge of two ^5^. Based on the angular momentum conservation law, the conversion from the LCP incident beam to the RCP output beam is induced by spin-to-orbital angular momentum conversion, where the spin angular momentum of the incident beam is converted to the orbital angular momentum (OAM) of the RCP light scattered from a single particle of interest. Based on this equation, the conversion efficiency is dependent on the value of $\left|t_{p}\cos\theta-t_{s}\right|$; the higher this value is, the higher the conversion efficiency. According to the calculated curves shown in Figure 1f, the 1D PC substrate increases the coefficient $\left|t_{p}\cos\theta-t_{s}\right|$.

**Section** **3: The design principle of a 1D PC and its dependence on particle size**

A 1D PC is designed to enhance the spin-to-orbit angular momentum conversion of scattered light from single particles or to enhance the depolarization effect of scattered light. To design a proper 1D PC substrate, the angular-dependent distribution of the scattered light from single particles under illumination by a left-circularly polarized (LCP) beam should first be calculated. The particle is placed on a bare glass substrate, and the scattered light will be mainly from the LCP components. This distribution varies with the size of the particles. As shown in Figure S3a-c, when the size of the particle is larger than the half-wavelength of the incident beam, such as for polystyrene nanoparticles with a diameter of 500 nm placed on a glass substrate, the angular-dependent distribution is mainly located inside the critical angle. Conversely, when a particle is very small, such as an Au nanoparticle with a diameter of 20 nm, the angular-dependent distribution is mainly outside of the critical angle.

Based on its angular-dependent distribution, the thickness of each layer of the 1D PC substrate is tuned to modify the transmissivity of the scattered light through the 1D PC substrate so that the term $\left|t_{p}\cos\theta-t_{s}\right|$ can reach a large value. Here, $\theta$ is the incident angle of the scattered light. $t_{p}$ and $t_{s}$ are the electric field transmissivities for *p*- and *s*-polarized scattered light through the 1D PC substrate, respectively. In this case, when the scattered light passes through this properly designed 1D PC substrate, it will undergo polarization conversion from LCP to RCP. Thus, the depolarization effect of the scattered light will be enhanced. During this conversion from LCP to RCP, the scattered light emitted from the 1D PC substrate will carry an orbital angular momentum to meet the angular momentum conservation law. The proper design of a 1D PC substrate can enhance this conversion efficiency.

It should be noted that one 1D PC substrate is not only suitable for one particle size but also suitable for a range of particle sizes. For example, for nanoparticles with a diameter less than or approximately half the wavelength of the incident beam (such as nanoparticles with a diameter less than 100 nm or 200 nm), the angular-dependent distribution of the scattered light from the single particles changes slightly with the particle size, and one properly designed 1D PC substrate can suffice for experiments involving these sizes. As demonstrated in our experiment, one 1D PC can be used for tracking single nanoparticles with diameters of 20 nm, 50 nm, and 100 nm. (Figure 5 and S11).

When the size of the particle is much larger, such as larger than or nearly one wavelength of incident beam (such as polystyrene beads at 500 nm diameter), another 1D PC substrate should be designed because the angular-dependent distribution of the scattered light from the larger particle is quite different from that of much smaller particles (as shown in the comparisons between Figure 1b and Figure S3).

**Section 4: Detailed descriptions of the data analysis procedures for the captured iSCAT microscopy images**

The spatial position of the two lobes in the double-helix pattern was always determined by using two different methods: weighted centroid calculation and least-squares Gaussian fit^6-10^. The first method determines the position of the double-helix lobes by simple threshold processing and weighted centroid calculation, which is simpler and more convenient than the second method. The second method provides higher position accuracy, but its robustness is poor because it requires a symmetrical shape.

For single polystyrene particles with a diameter of 500 nm, the image contrast is high, as shown in Figure S6a. We directly used threshold processing and weighted centroid calculations to determine the centers of the two lobes and the angular orientation of the two lobes (Figure S6b), which denotes the axial position of the particle. For small nanoparticles such as a single Au nanoparticle with a diameter of approximately 20 nm, the contrast of the DH-PSF iSCAT microscopy image is low, and the nonuniform background hinders the determination of the centers of the two lobes, as shown in Figure 5b. Background subtraction was performed on the raw image, and the least squares Gaussian fitting method was subsequently used to determine the centers of the two lobes, as shown in Figure S6c and Figure S6d, because the two lobes of DH-PSF are more symmetrical when the nanoparticles are small. The two lobes rotate with the axial location of the nanoparticle, so we can estimate the axial location of this particle through the calibration curve of the angle *vs.* axial location, as shown in Figure 3d and 3h and Figure 5d. The X and Y positions of the nanoparticle can be derived through the midpoint of the center of the two lobes, and the Z position can be derived by mapping the angular orientation of the two lobes to its axial location.

**Section 5: The estimation of localization precision and the influence of aberration on calibration**

We recorded the 3D positions of three polystyrene particles with a diameter of 500 nm at different positions 100 times and corrected the background drift by subtracting the spatial position of a reference particle. For example, the histograms of the spatial coordinates when the particles were located on the plane (Z = 0.2 μm) are shown in Figure S7a-7c, from which we determined the standard deviations of the particle positions. The standard deviations were estimated as $(\sigma_{x}, \sigma_{y}, \sigma_{z}) = (1.5nm, 1.4 nm, 5.5 nm)$, which can be considered the localization precision of a single measurement ^11, 6^. The localization precision within the axial working range of approximately 2 μm near the focal plane is shown in Figure S7d, and the average localization precision within the entire axial working range is $(\sigma_{x}, \sigma_{y}, \sigma_{z}) = (1.4 nm, 1.6 nm, 8.2 nm)$. In the future, the localization precision can be further improved through a more stable optical system, more precise drift correction and other improvements.

In the calibration experiment, the calibration curve for DH-PSF was generated by moving the substrate and its attached particles through the Piezo stage and obtaining images at different axial positions. On the other hand, in the single-particle motion experiment in solution, the focal point and substrate remain fixed, and the particles move relative to the focal plane and substrate. These two situations have different defocusing aberrations. To demonstrate this difference, an optical tweezer was used in the experimental setup (Figure S8a) to trap this single particle in a water solution so that this particle could be nearly fixed and its spatial location would not change. Optical tweezers were obtained by using another laser beam at a wavelength of 671 nm. This laser beam was focused by the imaging objective, and its focal point was controlled by a spatial light modulator (SLM) that can modulate the spatial phase distribution of this laser beam through the use of a phase hologram. The focal point of the trapping laser beam was set above the substrate and inside the water solution. The trapped single particle was fixed at the focal point of the 671 nm wavelength laser beam.

At the same time, a Piezo stage was used to tune the axial position of the 1D PC substrate. By using this method, the axial distance between this single particle and the substrate can be precisely controlled. In the experiment, we precisely tuned this distance between the particle and the substrate by axially moving the Piezo stage in the range of 2 μm, as shown in Figure S8a. At each axial position Z (from 0 μm to 2 μm) of the substrate, a microscopy image was recorded, as shown in Figure S8b. During the recording of the images, the imaging objective is fixed; thus, the relative position of the single particle to the focal plane of the imaging objective is not changed. The orientations of the two lobes on DH-PSF were nearly the same, as shown in Figure S8b. The value of this particle’s axial position derived from the two-lobe angular orientation in the case of Z = 2 μm is only approximately 130 nm shifted from that in the case of Z= 0 μm, which is nearly consistent with the calculated value reported in reference ^12^. The curve of the shifted value *vs.* *Z* is plotted, as shown in Figure S8c. This kind of shift is mainly due to the different imaging aberrations between the experimental results for deriving the calibration curves and the experimental results for the motion of a single particle in solution. A slightly shifted value means that the difference is not very large, especially in the case of short axial-range tracking, and this difference can be considered when refining the calibration curves for higher position accuracy.

As reported in References^13, 12^, the influence of imaging aberration on the axial positioning of DH-PSFs is less significant than that of standard PSFs and 3D PSF engineering, such as astigmatism PSF and saddle point PSF. DH-PSF has better robustness and a smaller axial positioning error for the following reasons. For microscopy with a standard PSF, the axial location of a single particle can be derived from the contrast and size of the diffraction-limited Airy spot, which will change when the particle is away from the focal plane. The imaging aberration will have a stronger influence on this change. On the other hand, for microscopy with a DH-PSF, the axial position of a single particle is derived from the angular orientation of the two lobes, which is less affected by image aberration.

**Section 6: Theoretical mechanism of the axial location-dependent point spread function of iSCAT microscopy**

The intensity of the particle image $I$ is the sum of the transmitted background, the signal, and their interference, which can be written as

|  |  | (**S5.1**) |
| --- | --- | --- |

where $E_{t}$, $E_{s}$, and are the transmitted light, the forward scattered light of the particle, and their relative phase difference, respectively.

The transmitted light is a weakly focused Gaussian beam, approximately a plane wave, and the phase of transmitted light can be expressed as:

|  |  | (**S5.2**) |
| --- | --- | --- |

where $R_{t}(z)$ is the spherical wavefront of the transmitted light, $\arctan\frac{z}{z_{0}}$ is the Gouy phase and $z_{0}$ is the Rayleigh range of the transmitted Gaussian beam.

The light scattered by a particle has a second-order phase vortex, which is approximately a second-order Laguerre Gaussian beam ^14-16, 6^. The phase of scattered light can be written as

|  |  | (**S5.3**) |
| --- | --- | --- |

where  is the second-order phase vortex, $R_{s}(z)$ is the spherical wavefront, $\arctan\frac{z}{z'_{0}}$ is the Gouy phase and $z'_{0}$ is the Rayleigh range of the Laguerre Gaussian beam.

Hence, the total phase difference can be written as:

|  |  | (**S5.4**) |
| --- | --- | --- |

In the vicinity of the focus, as the axial position of the particle changes, the Gouy phase shifts. Thus, the two lobes generated by interference between scattered light and transmitted light rotate around the optical axis (Z-axis). In areas far from the focus (such as Z = -15 μm or Z = 15 μm), the spherical wavefronts of the scattered light above and below the focal plane are opposite; as a result, the rotation directions of the spiral pattern handedness are reversed (as shown in Figure S5).

**References for SI reference citations**

1. Yang, J. J., Hugonin, J. P. & Lalanne, P. Near-to-Far Field Transformations for Radiative and Guided Waves. *Acs Photonics* **3**, 395-402 (2016).

2. Török, P., Higdon, P. D. & Wilson, T. On the general properties of polarised light conventional and confocal microscopes. *Optics Communications* **148**, 300-315 (1998).

3. Foreman, M. R. & Török, P. Computational methods in vectorial imaging. *Journal of Modern Optics* **58**, 339-364 (2011).

4. Bliokh, K. Y. et al. Spin-to-orbital angular momentum conversion in focusing, scattering, and imaging systems. *Optics Express* **19**, 26132-26149 (2011).

5. Long, O. Y. et al. Isotropic topological second-order spatial differentiator operating in transmission mode. *Optics Letters* **46**, 3247-3250 (2021).

6. Pavani, S. R. P. et al. Three-dimensional, single-molecule fluorescence imaging beyond the diffraction limit by using a double-helix point spread function. *Proceedings of the National Academy of Sciences of the United States of America* **106**, 2995-2999 (2009).

7. Thompson, M. A. et al. Localizing and tracking single nanoscale emitters in three dimensions with high spatiotemporal resolution using a double-helix point spread function. *Nano Letters* **10**, 211-218 (2010).

8. von Diezmann, A. et al. Correcting field-dependent aberrations with nanoscale accuracy in three-dimensional single-molecule localization microscopy. *Optica* **2**, 985-993 (2015).

9. Wang, F. et al. Three-dimensional diffusion coefficient measurement by a large depth-of-field rotating point spread function. *Applied Optics* **60**, 10766-10771 (2021).

10. Wang, F. et al. Double helix point spread function with variable spacing for precise 3D particle localization. *Optics Express* **31**, 11680-11694 (2023).

11. Thompson, R. E., Larson, D. R. & Webb, W. W. Precise nanometer localization analysis for individual fluorescent probes. *Biophys J* **82**, 2775-2783 (2002).

12. Siemons, M. E., Kapitein, L. C. & Stallinga, S. Axial accuracy in localization microscopy with 3D point spread function engineering. *Optics Express* **30**, 28290-28300 (2022).

13. Ghosh, S. & Preza, C. Characterization of a three-dimensional double-helix point-spread function for fluorescence microscopy in the presence of spherical aberration. *Journal of Biomedical Optics* **18**, 036010 (2013).

14. Schechner, Y. Y., Piestun, R. & Shamir, J. Wave propagation with rotating intensity distributions. *Physical Review E* **54**, R50-R53 (1996).

15. Piestun, R., Schechner, Y. Y. & Shamir, J. Propagation-invariant wave fields with finite energy. *Journal of the Optical Society of America A* **17**, 294-303 (2000).

16. Pavani, S. R. & Piestun, R. Three dimensional tracking of fluorescent microparticles using a photon-limited double-helix response system. *Optics Express* **16**, 22048-22057 (2008).

**
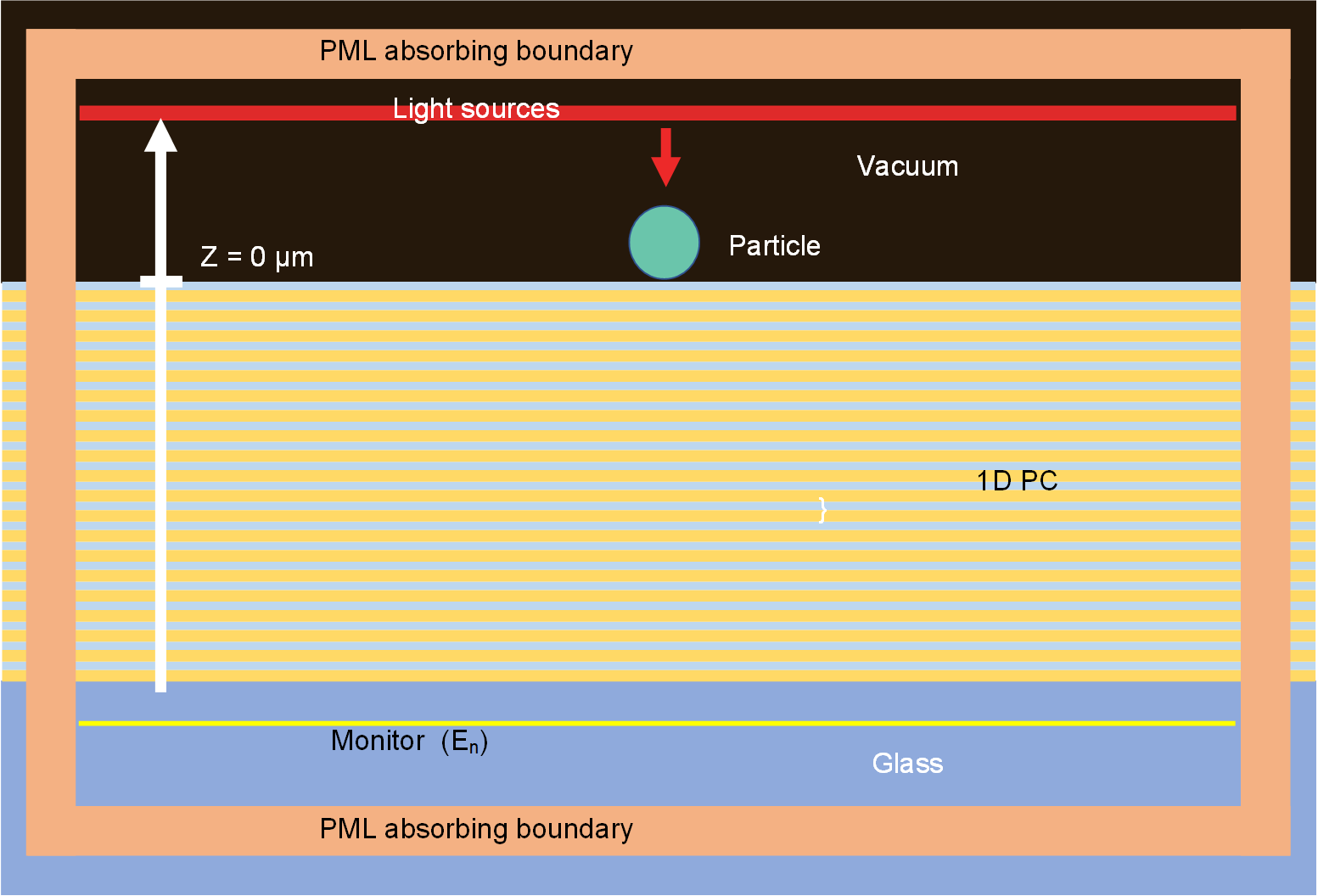
**

**Figure S1.** Schematic diagram of the simulation model for calculating the near-field distribution of the iSCAT signals.


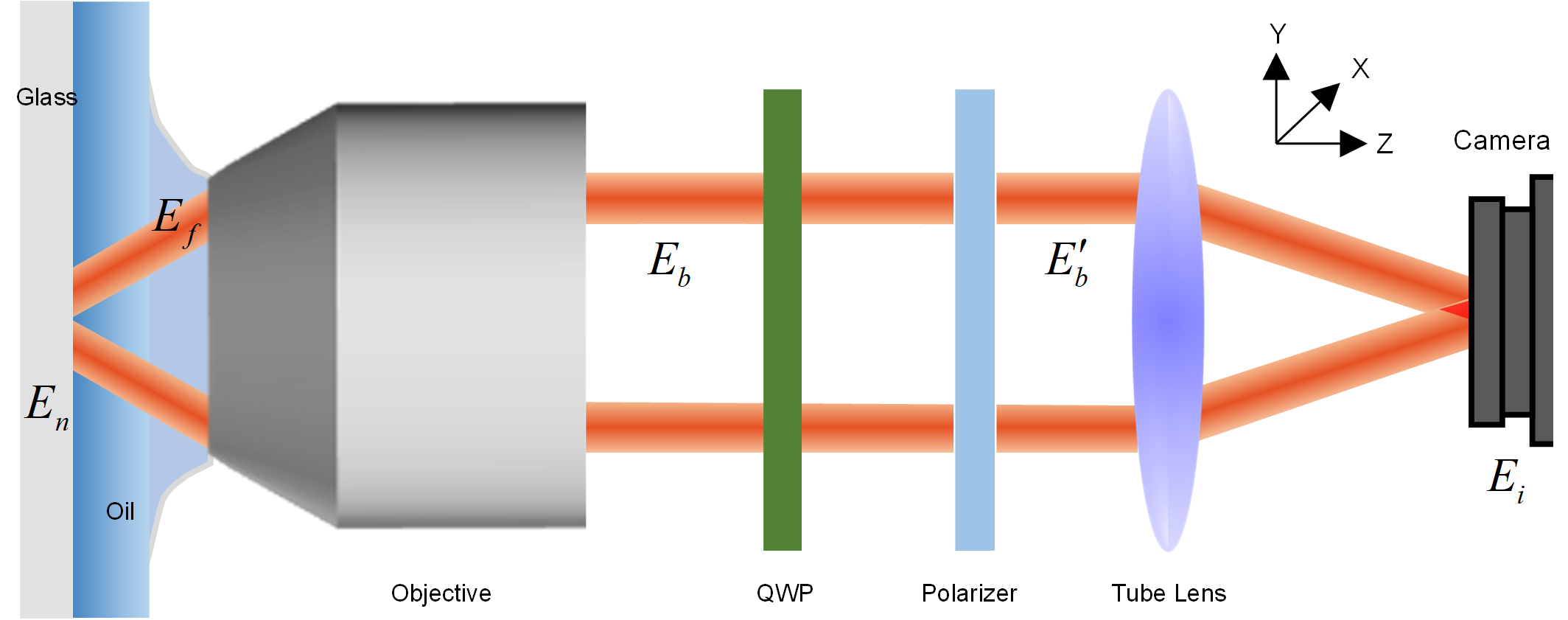


**Figure S2.** Schematic diagram of the simulation model for calculating the electric field distribution at the BFP and FFP of the iSCAT signals.


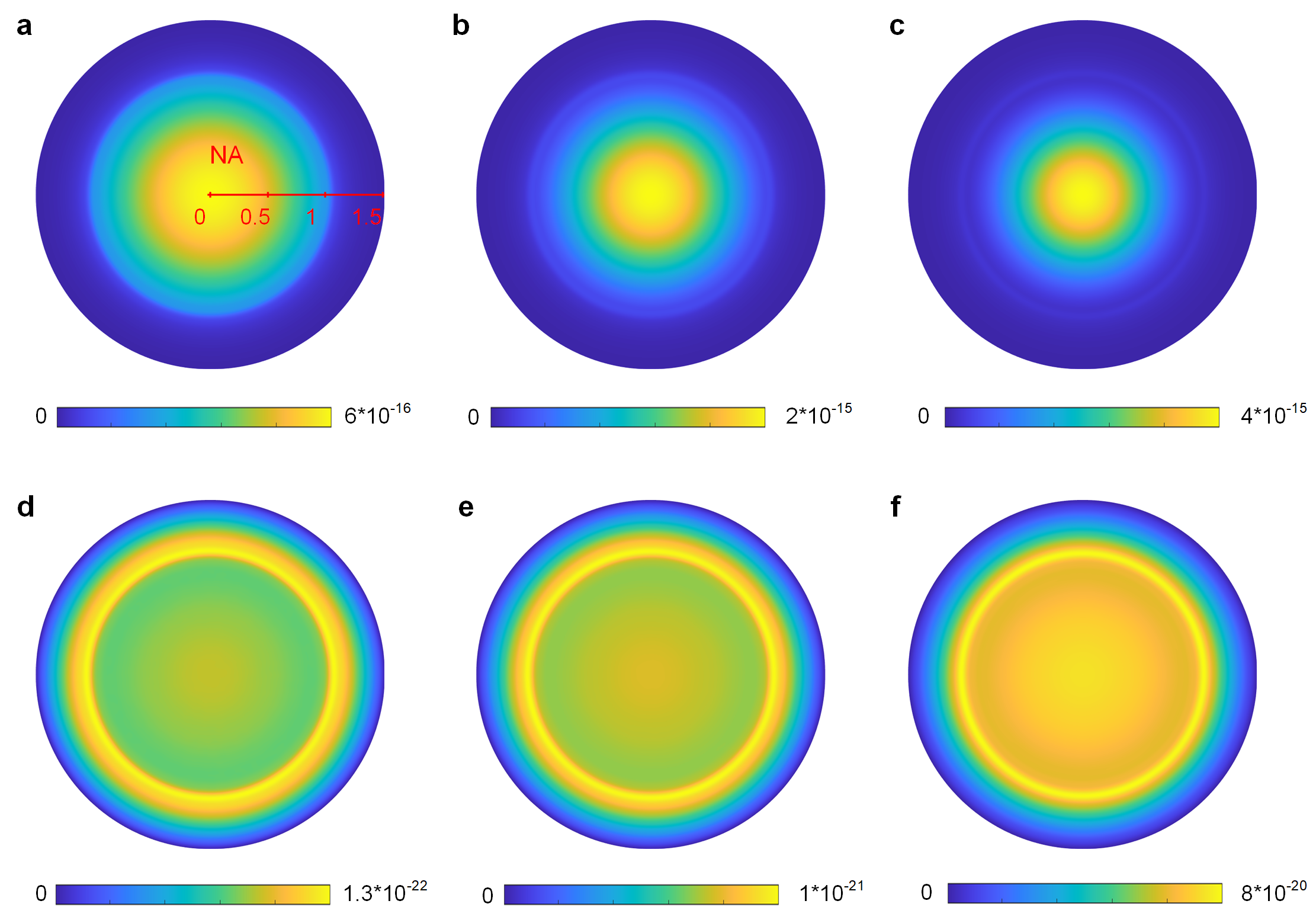


**Figure S3.** **The calculated angular-dependent distributions of the scattered light from single particles.** The single particles were placed on glass substrates. (a, b, c) Calculated far field angular-dependent distributions of scattered light from a single microparticle, where (a) is a polystyrene particle with a diameter of 400 nm, (b) is a polystyrene particle with a diameter of 500 nm, and (c) is a polystyrene particle with a diameter of 600 nm. (d, e, f) Calculated far-field angular-dependent distributions of scattered light from a single nanoparticle, where (d) is a gold nanoparticle with a diameter of 20 nm, (e) is a polystyrene nanoparticle with a diameter of 50 nm, and (f) is a polystyrene nanoparticle with a diameter of 100 nm.


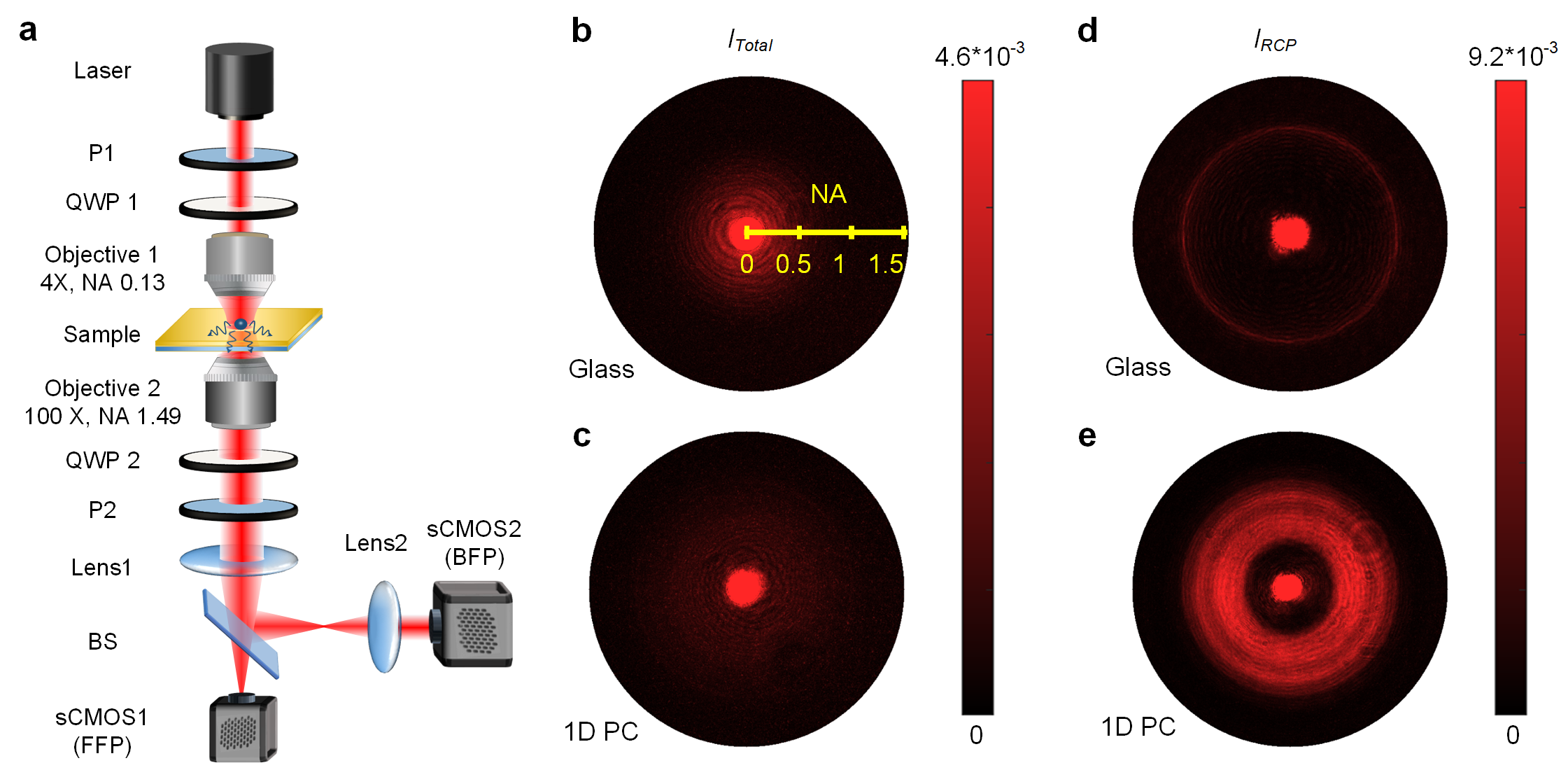


**Figure S4. The measurements of the front focal plane (FFP) and back focal plane (BFP) images**. (a) Schematic of the experimental setup of the proposed iSCAT microscope. P1 and P2, polarizers 1 and 2. QWP1 and QWP2, quarter wave plates 1 and 2. BS, beam splitter. The sample consists of a single particle on either glass or a 1D PC substrate. (b, c) The measured experimentally angular-dependent distributions of the total electric field intensity (*I_Total_*) from a single particle when the particle was placed on a glass (b) or 1D PC substrate (c); here, the combination of QWP2 and P2 was removed from the optical setup. (d, e) The measured experimentally angular-dependent distributions of the RCP light (*I_RCP_*) for the particle on the glass (d) or 1D PC substrate (e), where the combination of QWP2 and P2 was placed before the camera to allow only RCP light to pass through. The appearance of the center bright spots (d, e) can be attributed to the depolarization effects during the imaging process and the direct transmission of the illumination LCP light that has not been fully blocked by the combination of QWP2 and P2.


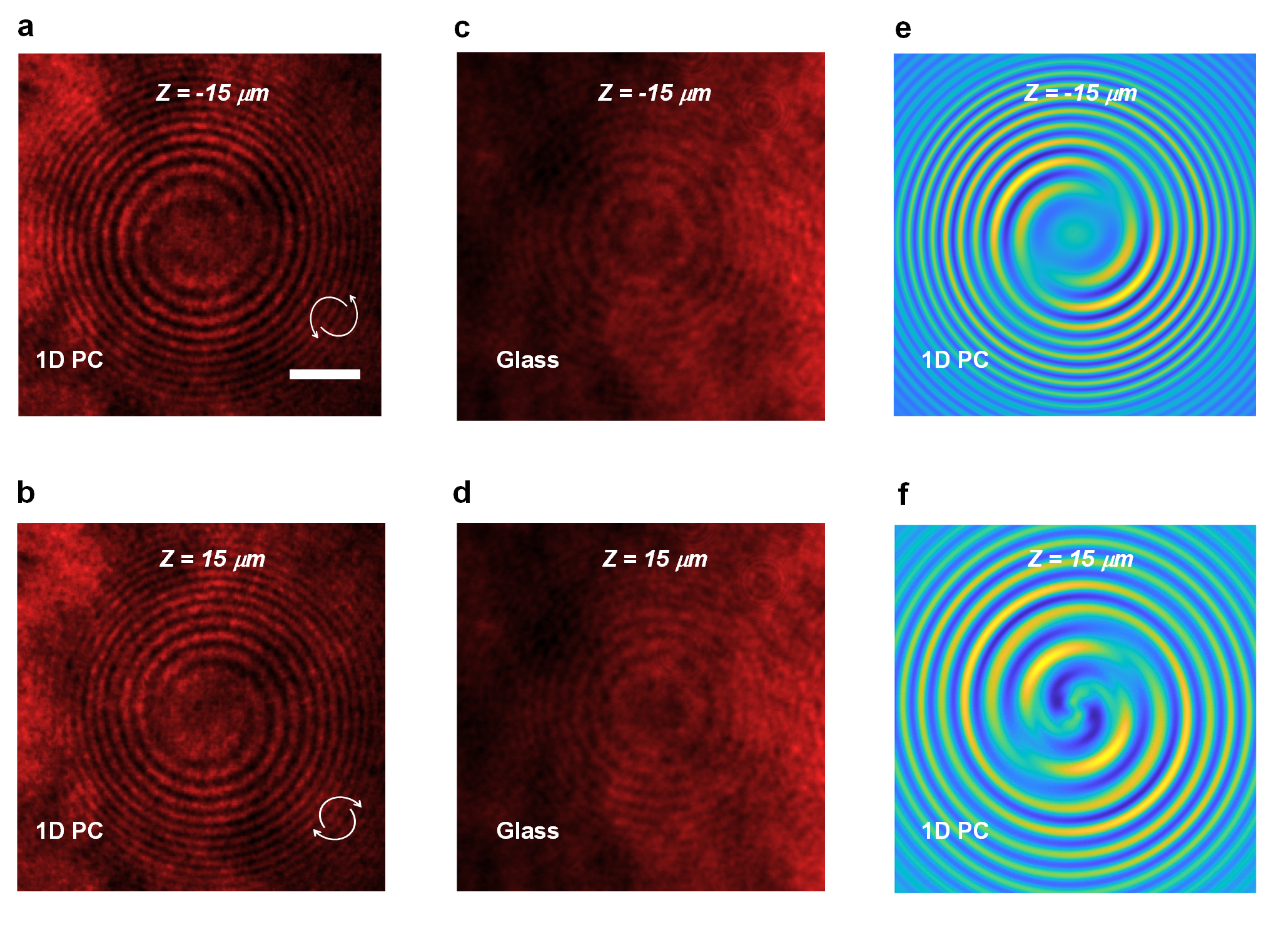


**Figure S5. Images of a single particle located far from the focal plane of the objective lens.** (a-d) iSCAT images when the sample was placed on a 1D PC substrate or glass substrate below (Z = -15 μm) or above (Z = 15 μm) the focal plane of the imaging objective. (e, f) Simulated out-of-focal plane iSCAT images at 15 μm above or below the focal plane. Scale bars, 5 µm.

**
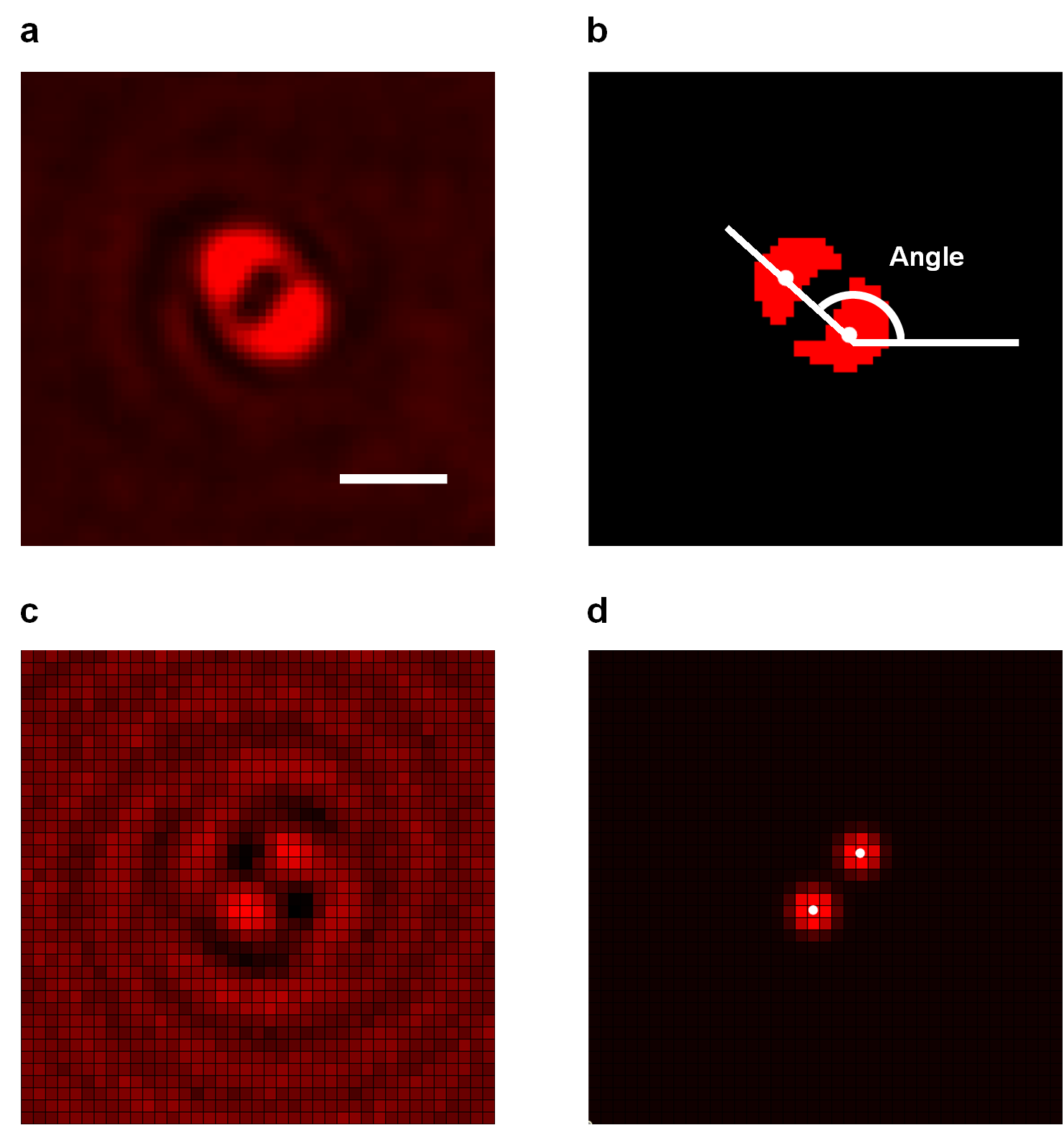
**

**Figure S6. Calibration method for a single microparticle located within a range of 2 μm near the focal plane**. (a) The original DH-PSF iSCAT image of 500 nm polystyrene nanoparticles. (b) The image used a weighted centroid calculation to derive the center of the two lobes and the value of the angle. (c) The ratiometric image of a 20 nm gold particle obtained by subtracting the background. (d) The image used the double Gaussian fitting method to derive the center of the two lobes and the value of the angle. Scale bar, 1 µm.


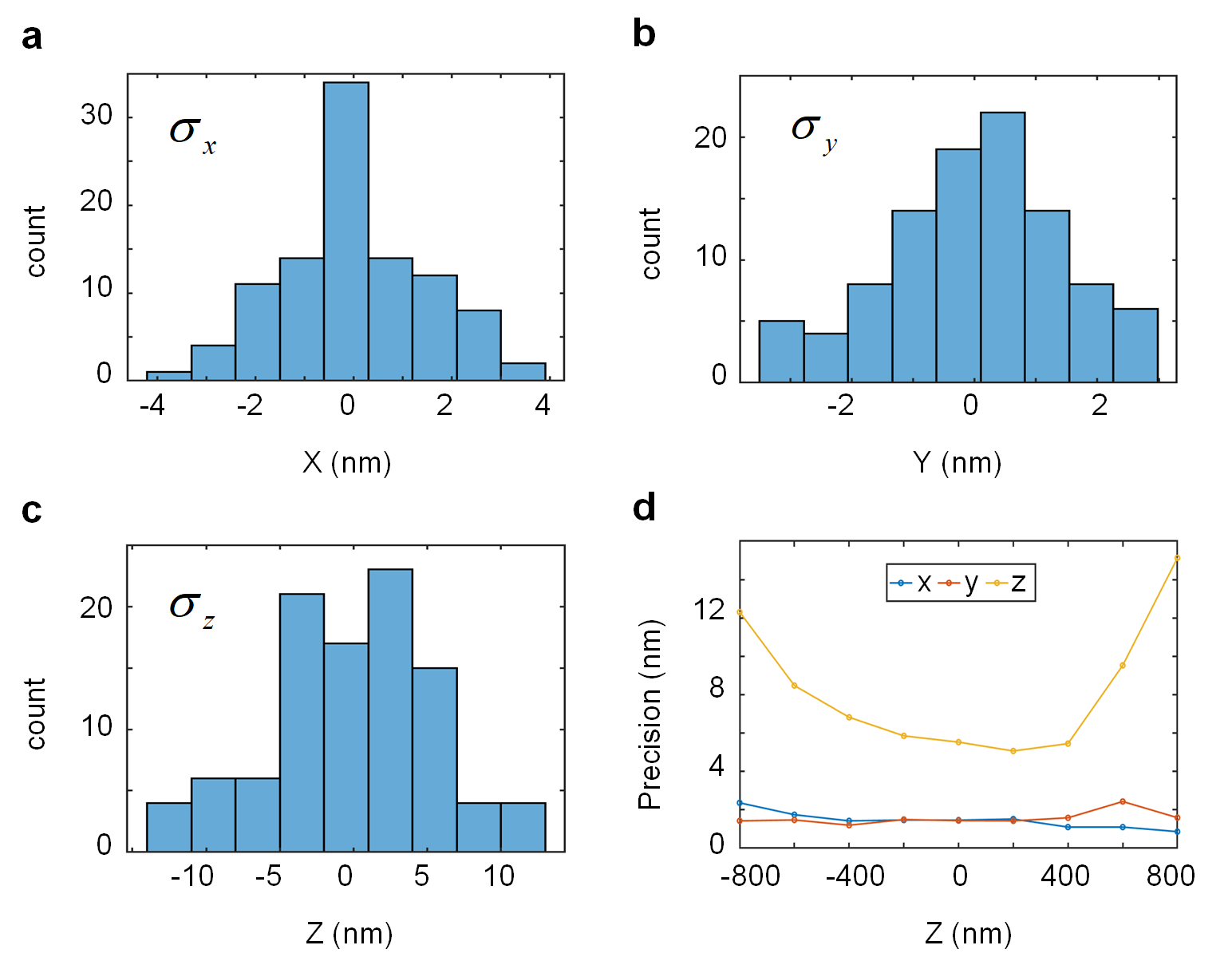


**Figure S7. Location precision of the DH-PSF iSCAT method.** (a, b, c) The histogram of 100 localizations of 500 nm polystyrene particles at the axial position (Z = 0.2 μm) in the X, Y, and Z directions. (d) The localization precision in the X, Y, and Z axes within an axial range of approximately 2 μm near the focal plane.

**
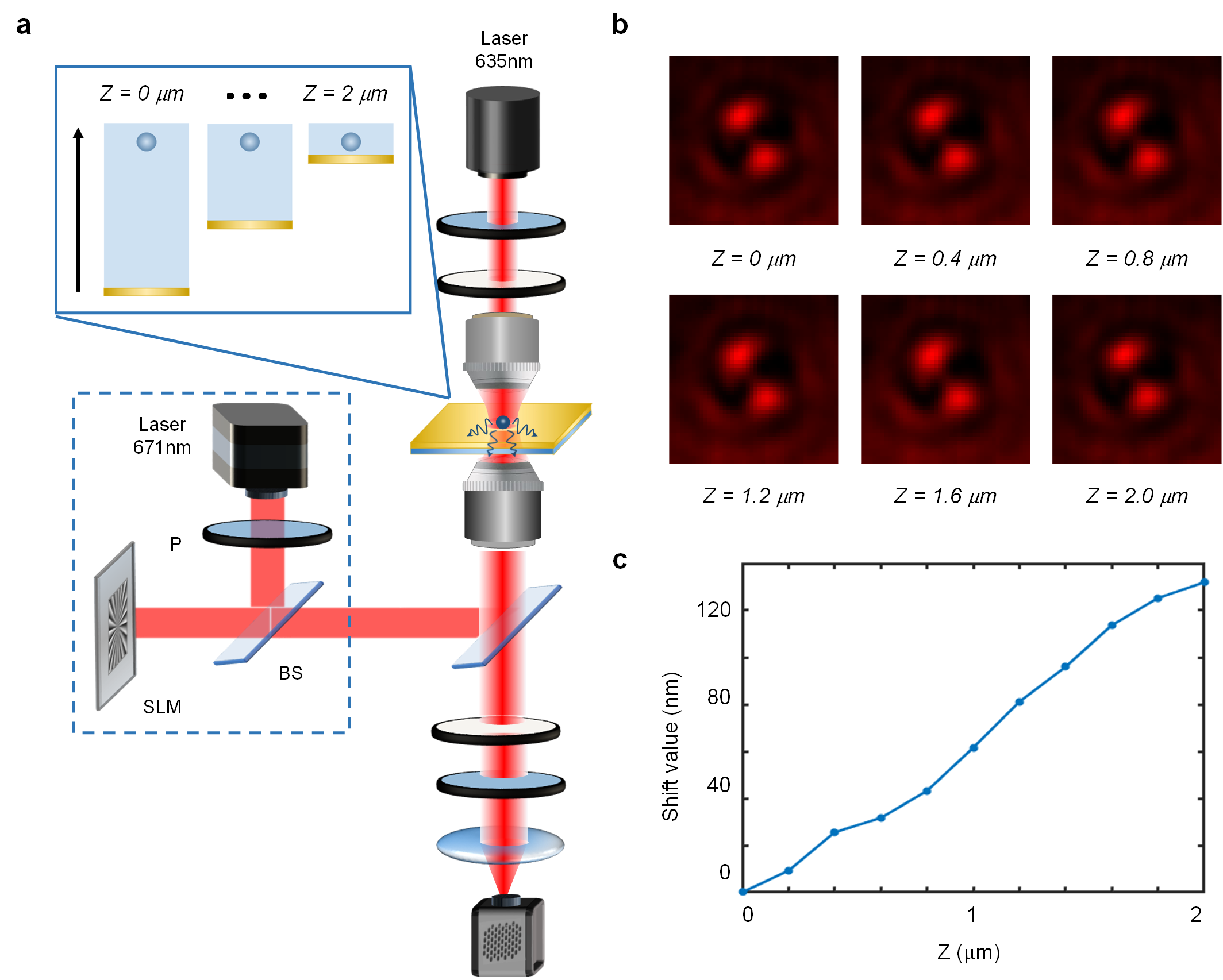
**

**Fig****ure S8.** **The influence of imaging aberration on axial position calibration.** (a) Schematic of the experimental setup for DH-PSF iSCAT microscopy with an optical tweezer module. P, polarizer; BS, beam splitter; SLM, spatial light modulator. (b) A series of DH-PSF iSCAT images were captured when the axial position of the substrate was tuned through a Piezo stage. The axial position of the substrate Z changes from 0 μm to 2 μm. The single particle was trapped with optical tweezers, and then its position was fixed. (c) The curve of the shifted value *vs.* the axial position Z of the substrate. The shift value is defined as the difference between the axial position of the single particle derived from the DH-iSCAT image (Z=0 μm) and the axial position of the single particle derived from the other DH-iSCAT images (Z range from 0 to 2 μm).


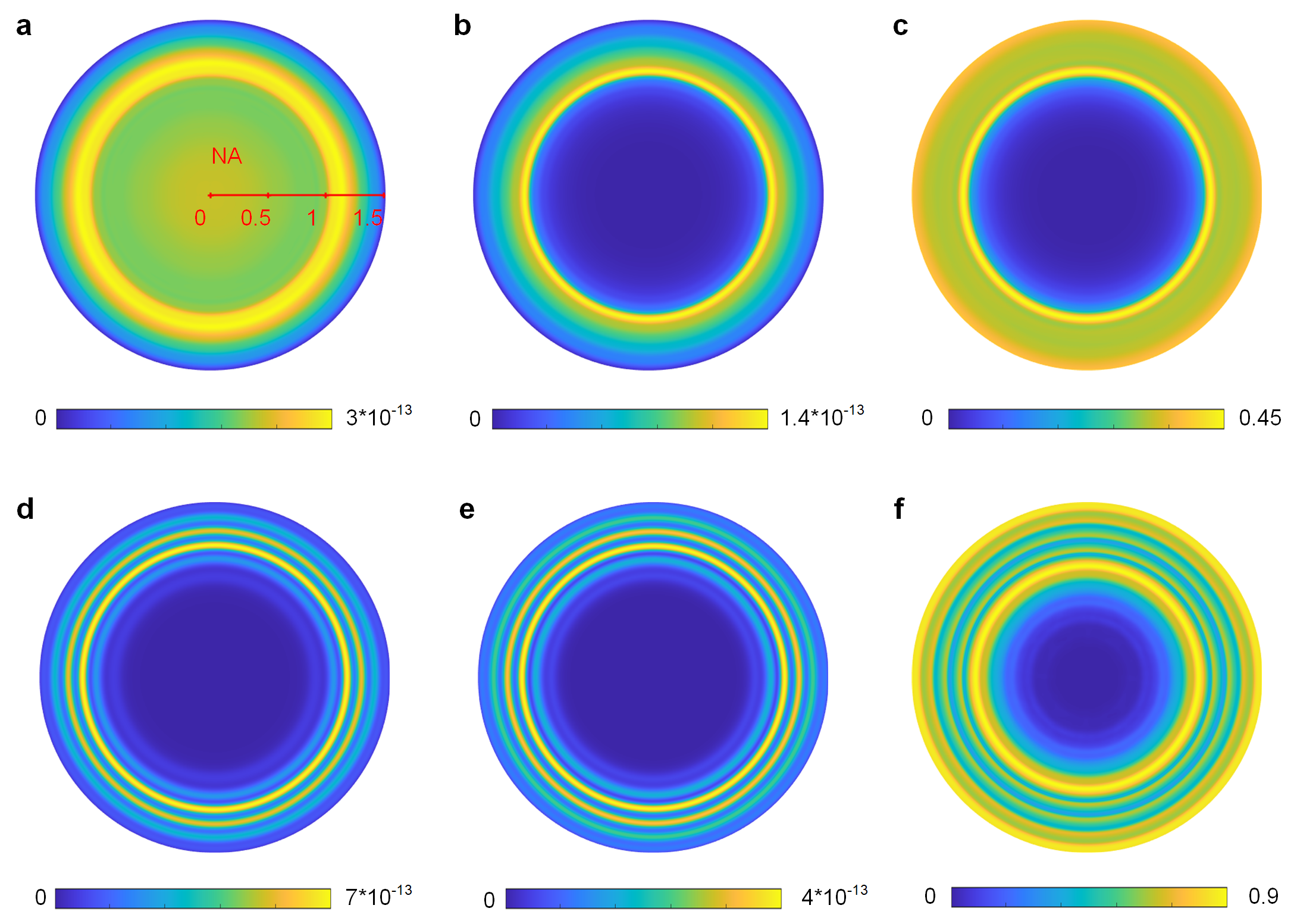


**Fig****ure S9. The calculated radiation field from an electric dipole placed on either the glass substrate or the 1D PC substrates**. (a, b) Calculated far-field angular-dependent distributions of total radiation light and RCP-radiation light from an electric dipole placed on a glass substrate; (c) ratio between (b) and (a). (d, e, f) The same electric dipole was placed on a dielectric 1D PC substrate.


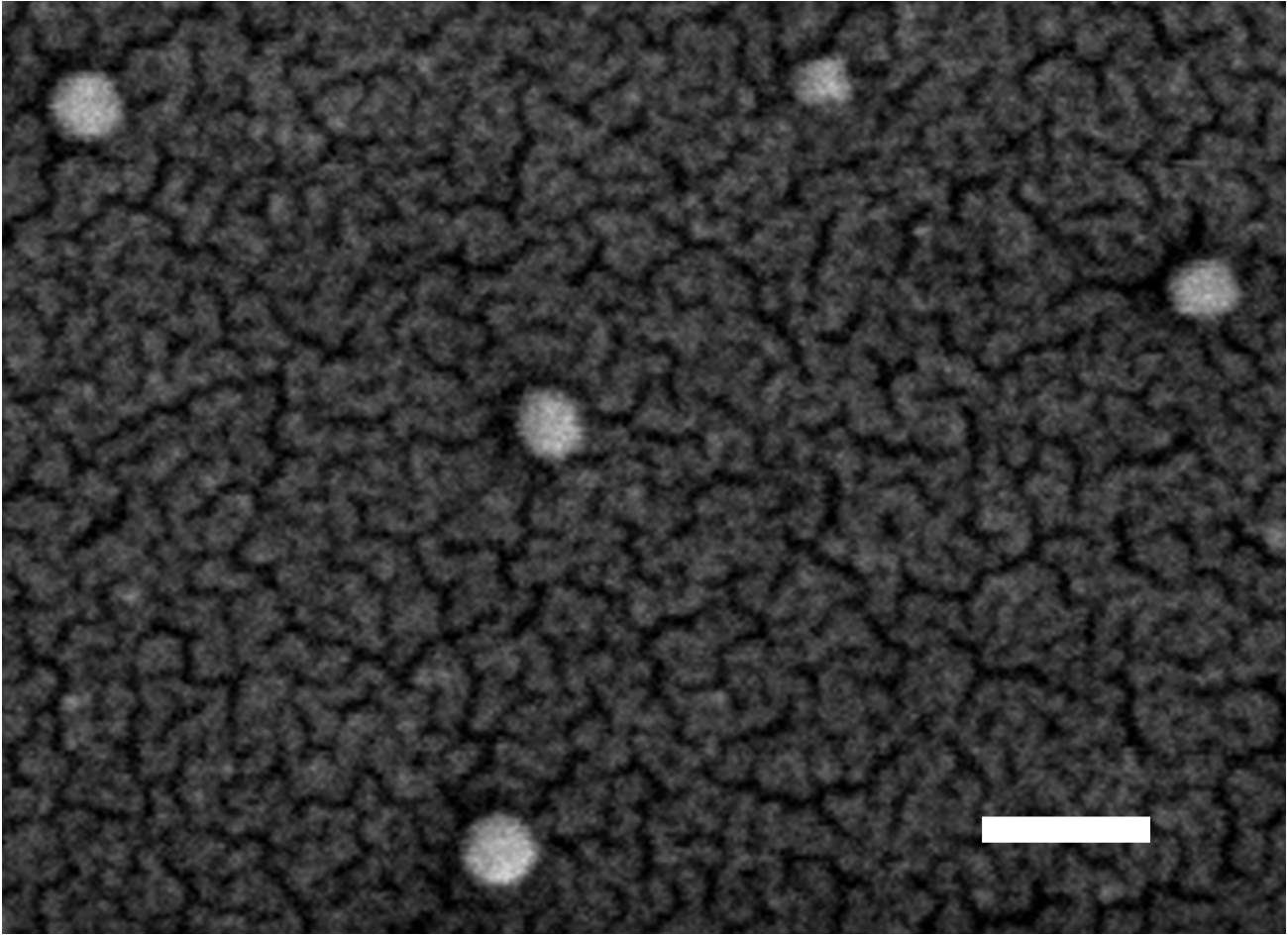


**Figure S10.** **SEM image of gold nanoparticles with a diameter of 20 nanometers**. Scale bars, 50 nm.


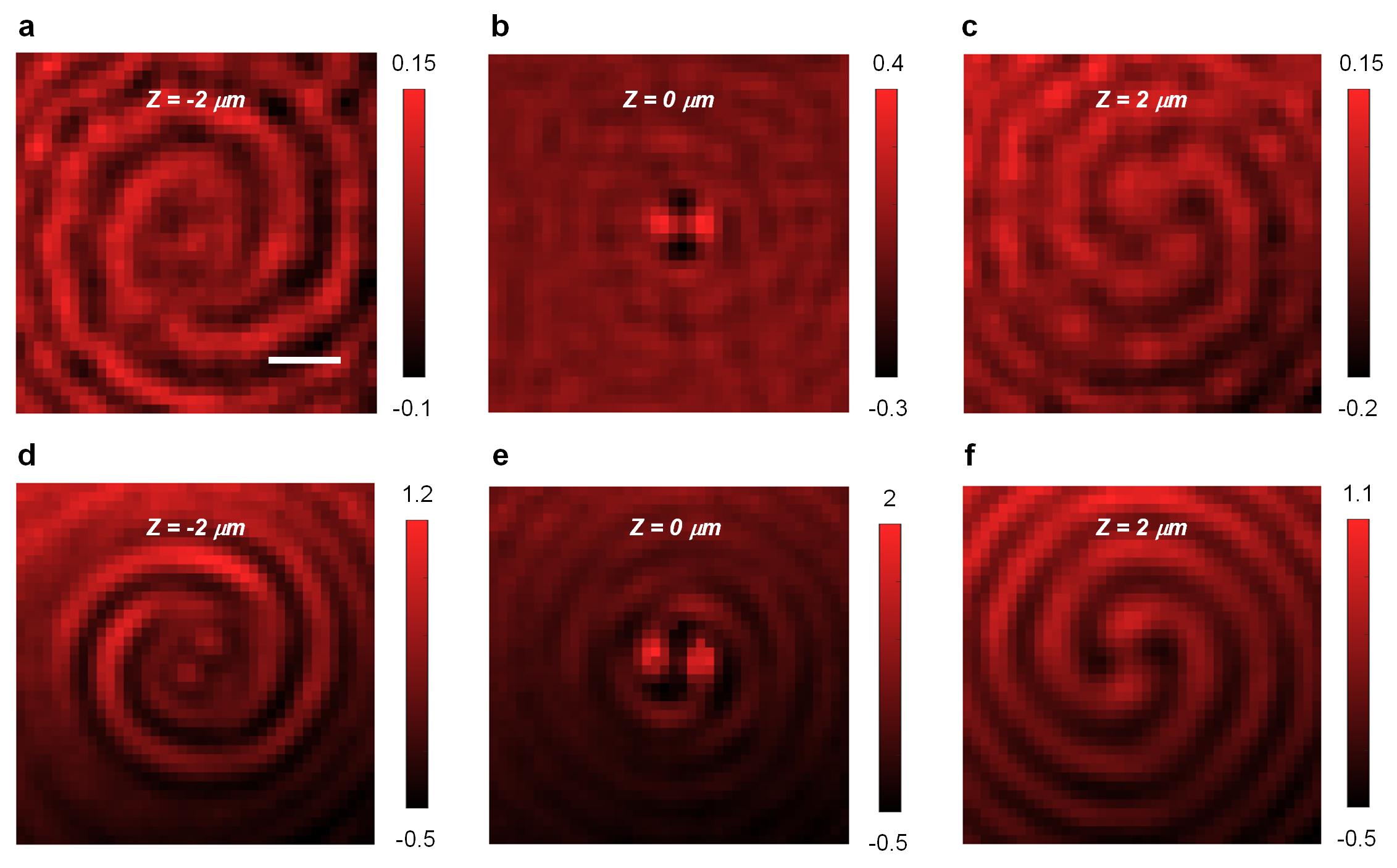


**Figure S11.** **DH-PSF iSCAT images of single polystyrene nanoparticles with diameters of 50 nm and 100 nm.** (a-c) Resulting ratiometric image of polystyrene nanoparticles with a diameter of 50 nanometers obtained by subtracting the background when the particle is below (Z = -2 μm), on (Z = 0 μm) or above (Z = 2 μm) the focal plane of the imaging objective. (d-f) Resulting ratiometric image of polystyrene nanoparticles with a diameter of 100 nanometers obtained by subtracting the background when the particle is below (Z = -2 μm), on (Z = 0 μm) or above (Z = 2 μm) the focal plane of the imaging objective. Scale bars, 1 µm. The contrast is defined in the Methods section.


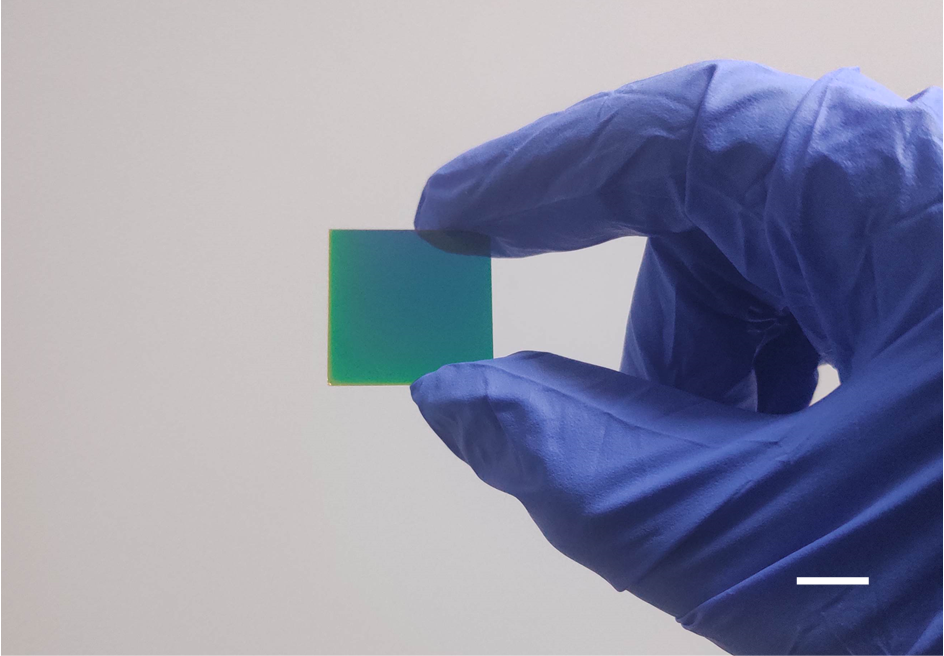


**Fig****ure S12.** Photograph of a fabricated all-dielectric 1D PC. Scale bars, 1 cm.

**Movie 1:** Demonstration of the rotation of the double helix pattern with the axial location of the particle (near the focal plane) and the formation of the calibration curve (numerical simulation results).

**Movie 2:** Demonstration of the rotation of the double helix pattern with the axial location of the particle (near the focal plane) and the formation of the calibration curve (experimental results).

**Movie 3:** 3D trajectories of the particle attached to the filament stub of a living bacterium.

**Movie 4:** 3D diffusion trajectories of single gold nanoparticles recorded by DH-PSF iSCAT microscopy.
